# Supplementary material for: Death within 1 year among emergency medical admissions to Scottish hospitals: incident cohort study
Source: BMJ Open. 2018 Jun 30;8(6):e021432. doi: 10.1136/bmjopen-2017-021432 (PMC6042622; doi:10.1136/bmjopen-2017-021432)
Supplement: Supplementary file 1 [file bmjopen-2017-021432supp001.pdf]

Supplementary Table: Cox regression analysis for mortality at one year in cancer and non cancer patients

	Cancer patients					Non-cancer patients				
	**No. of deaths**	**No. of patients**	**Crude mortality rate (per 1,000)**	**Adjusted hazard ratio (95%CI)**	**p-value**	**No. of deaths**	**No. of patients**	**Crude mortality rate (per 1,000)**	**Adjusted hazard ratio (95%CI)**	**p-value**
**Age**										
<60	139	300	463	1	-	170	3,615	47	1	-
60-64	64	118	542	1.27 (0.94-1.71)	0.117	68	592	115	2.52 (1.90-3.34)	<0.001
65-69	124	212	585	1.38 (1.08-1.76)	0.009	110	714	154	3.40 (2.67-4.32)	<0.001
70-74	147	258	570	1.37 (1.09-1.73)	0.008	150	808	186	4.15 (3.33-5.18)	<0.001
75-79	139	264	527	1.20 (0.95-1.52)	0.133	212	911	233	5.23 (4.27-6.40)	<0.001
80-84	125	200	625	1.51 (1.19-1.93)	0.001	281	1,013	277	6.67 (5.51-8.08)	<0.001
85+	132	213	620	1.56 (1.23-1.98)	0.000	485	1,259	385	10.16 (8.50-12.13)	<0.001
**Sex**										
Females	381	742	513	1	-	757	4,721	160	1	-
Males	489	823	594	1.23 (1.07-1.41)	0.003	719	4,191	172	1.33 (1.20-1.47)	<0.001
**Deprivation**										
SIMD 5 = least	140	255	549	1	-	203	1,141	178	1	-
SIMD 4	175	293	597	1.15 (0.92-1.44)	0.208	222	1,374	162	0.99 (0.82-1.20)	0.949
SIMD 3	151	269	561	1.09 (0.86-1.37)	0.486	260	1,575	165	1.00 (0.84-1.21)	0.967
SIMD 2	206	380	542	1.01 (0.82-1.26)	0.911	368	2,098	175	1.11 (0.94-1.32)	0.230
SIMD 1 = most	198	368	538	1.02 (0.82-1.27)	0.856	423	2,724	155	1.16 (0.98-1.38)	0.083
**Emergency admission in previous year**										
No	306	617	496	1	-	513	4,793	107	1	-
Yes	564	948	595	1.31 (1.14-1.51)	<0.001	963	4,119	234	1.26 (0.91-1.73)	0.159
Time*Emergency	-	-	-	-	-	-	-	-	1.12 (1.04-1.21)	0.002

Estimates of hazard ratio for Emergency admission in the previous year for **all patients** at different points in the follow-up

Time (days)	Estimated hazard ratio	LCI	UCI
0	1.25	0.93	1.58
7	1.49	1.27	1.71
30	1.67	1.51	1.83
60	1.77	1.61	1.92
90	1.83	1.66	1.99
120	1.87	1.69	2.05
180	1.93	1.73	2.14
366	2.05	1.77	2.34

Estimates of hazard ratio for Emergency admission in the previous year for **non-cancer patients** at different points in the follow-up

Time (days)	Estimated hazard ratio	LCI	UCI
0	1.26	0.86	1.66
7	1.60	1.30	1.89
30	1.87	1.64	2.09
60	2.02	1.80	2.24
90	2.11	1.88	2.35
120	2.18	1.93	2.44
180	2.29	1.99	2.59
366	2.48	2.07	2.90
