# Supplementary material for: Death within 1 year among emergency medical admissions to Scottish hospitals: incident cohort study
Source: BMJ Open. 2018 Jun 30;8(6):e021432. doi: 10.1136/bmjopen-2017-021432 (PMC6042622; doi:10.1136/bmjopen-2017-021432)
Supplement: Author's manuscript [file bmjopen-2017-021432.draft_revisions.pdf]

BMJ Open is committed to open peer review. As part of this commitment we make the peer review history of every article we publish publicly available.

When an article is published we post the peer reviewers' comments and the authors' responses online. We also post the versions of the paper that were used during peer review. These are the versions that the peer review comments apply to.

The versions of the paper that follow are the versions that were submitted during the peer review process. They are not the versions of record or the final published versions. They should not be cited or distributed as the published version of this manuscript.

BMJ Open is an open access journal and the full, final, typeset and author-corrected version of record of the manuscript is available on our site with no access controls, subscription charges or pay-per-view fees (http://bmjopen.bmj.com).

If you have any questions on BMJ Open's open peer review process please email info.bmjopen@bmj.com

BMJ Open

Death within one year among emergency medical admissions to hospital: incident cohort study.

Journal:	*BMJ Open*
Manuscript ID	bmjopen-2017-021432
Article Type:	Research
Date Submitted by the Author:	28-Dec-2017
Complete List of Authors:	Moore, Emily ; NHS Scotland National Services Division<br>Munoz-Arroyo, Rosalia; NHS Scotland National Services Division<br>Schofield, Lauren; NHS Scotland National Services Division<br>Radley, Alice ; Dumfries and Galloway Royal Infirmary, Medicine<br>Clark, David; University of Glasgow,, School of Interdisciplinary Studies,<br>Isles, Chris; Dumfries ,
Keywords:	emergency medical admissions, mortality, end of life care, anticipatory care plans

SCHOLARONE™
Manuscripts

Death within one year among emergency medical admissions to hospital: incident cohort study.

Emily Moore[1], Rosalia Munoz-Arroyo[1], Lauren Schofield[1] , Alice Radley[3], David Clark[2] and Chris Isles[3]
[1]NHS National Services Scotland, 1 South Gyle Crescent, Edinburgh EH12 9EB, [2]School of Interdisciplinary Studies, University of Glasgow, and [3]Department of Medicine, Dumfries and Galloway Royal Infirmary, Dumfries DG1 4AP

Emily Moore Information Analyst

Rosalia Munoz-Arroyo Principal Information Analyst

Lauren Schofield Senior Information Analyst

Alice Radley Core Medical Trainee

David Clark Professor of Medical Sociology

Chris Isles Consultant Physician

Correspondence
Prof Chris Isles
Medical Unit, Dumfries Infirmary,
Dumfries DG1 4AP
Chris.isles@nhs.net
07590 317255

For peer review only

Word count:

Key words: hospital inpatients; emergency medical admissions; mortality; anticipatory care planning; end of life care

Abstract

Objectives: To establish the likelihood of death within 12 months of admission to hospital; to examine the influence on survival of a cancer diagnosis made within the previous 5 years; and to compare mortality with that of the wider Scottish population.

Setting: Secondary care.

Participants: 10,477 patients admitted as an emergency to medicine in 22 Scottish hospitals in March 2015, 1,565 (14.9%) of whom had been given a cancer diagnosis in the previous five years.

Outcome measures: Mortality at intervals up to one year after the census admission.

Results: There were 2,346 (22.4%) deaths in the year following the census admission. Six hundred and ten patients died during the census admission (5.8% of all admissions and 26% of all deaths) while 1,736 died after the census admission (74% of all deaths). Malignant neoplasms (33.8%), circulatory diseases (22.5%) and respiratory disease (17.9%) accounted for almost three quarters of all deaths. Mortality rose steeply with age and was substantially higher at one year for patients aged 85 years and over compared to those who were under 60 years of age (adjusted hazard ratio 6.07, 95% CI 5.29 to 6.97, p<0.001) Cancer patients had higher mortality than patients without a cancer diagnosis (adjusted hazard ratio 3.79, 95% CI 3.48 to 4.13, p<0.001). Mortality was linearly related to the number of bed days occupied in the year before the census admission (p<0.001) but not to the number of admissions in that year. Age-sex standardized mortality was 110.4 (95% CI 104.4 to 116.5) for the cohort and 11.7 (95% CI 11.6 to 11.8) for the Scottish population, a 9.4 fold increase in risk.

Conclusion: These data may help identify patients admitted to hospital as medical emergencies who are at greatest risk of dying not only during admission but also in the following 12 months.

Word count 299

Strengths

- Patient cohort was drawn from all major teaching and general hospitals in Scotland
- Record linkage linked data from hospital system to national death registrations
- Incident rather than prevalent cohort study avoided length time bias

Limitations

- Results give likelihood of death for patient groups as defined but not for individual patients

For peer review only

Introduction

We have previously shown high 12 month mortality among Scottish hospital inpatients indicating that, for many individuals, admission to hospital is a sentinel event marking the transition to the last year of their lives (1,2). Nearly 1 in 10 patients died during admission, and almost 1 in 3 patients had died by a year later. This increased to nearly 1 in 2 for the over 85 age group. This information highlighted the need for clinicians to alter their approach to patient care in order to identify and address key end of life care needs (1,2). This realistic, patient-focussed approach has been widely advocated by the Gold Standards Framework (3), NICE (4), the General Medical Council(5), NHS England (6) and Health Improvement Scotland (7).

Despite these clear recommendations, many clinicians are reluctant to address end of life issues. A National Confidential Enquiry into Patient Outcome and Death (NCEPOD) review of the care of patients who died within 30 days of receiving systemic anti cancer therapy found that the decision to treat with chemotherapy was inappropriate in 19% cases. This raised questions as to whether cancer patients are given enough information about chemotherapy to enable them to make an informed consent to treatment (8). In a study of patients with metastatic lung and colorectal cancer, 69% and 81% of patients respectively were unaware that chemotherapy was highly unlikely to cure their cancer, again suggesting that clinicians are not comfortable with end of life care discussions (9). A survey of over 4000 US physicians found that one third would not discuss prognosis with a cancer patient who was asymptomatic but had only 4-6 months to live, preferring instead to wait until symptoms developed or there were no more treatments to offer (10).

Our previous study was of a prevalent rather than incident cohort which may have over-represented patients who had longer hospital stays. Likelihood of death was two times higher in medical patients than surgical patients, possibly reflecting the elective nature of most surgical admissions. We did not examine the influence of diagnosis, particularly a cancer diagnosis, as a predictor of death nor did we evaluate the relation between previous hospital admissions and mortality or the mortality risk of hospital inpatients compared to the wider population from which our patients were derived (1,2). The aims of the current study, therefore, were to examine an incident rather than a prevalent cohort; to focus on patients admitted as emergencies to medicine; to determine the impact on survival of a cancer diagnosis made within the previous 5 years; to assess whether the number or duration of previous admissions to hospital influenced mortality; and to compare mortality with that of that of an age and sex standardised Scottish general population.

For peer review only - http://bmjopen.bmj.com/site/about/guidelines.xhtml

Methods

We included only patients admitted to hospitals in Scotland where the most acute clinical activity occurs: large general hospitals (n=15) and teaching hospitals (n=7). On this occasion we limited our analyses to inpatients admitted as an emergency to medicine between March 18th and March 31st 2015. We defined an inpatient (rather than a day case) as a person who had a Scottish Morbidity Record (SMR01) episode with a discharge date either the day following the admission date, or later. We chose a 14 day census period in order to select sufficiently large numbers of admissions and deaths for robust statistical analysis.

In the event of an emergency readmission within the two week census period (n=216), we only counted the patient once. Thus our analysis is based on their first (or only) admission during this period. We refer to this as the census admission and the date of this admission as the census date. We classified patients as having a cancer diagnosis (any malignant neoplasm ICD-10 code C00-C97, excluding non-melanoma skin cancer (NMSC, C44)) (11) if they had an SMR01 record with a cancer diagnosis or a record in the Scottish Cancer Registry database (12) dated five years or less prior to their census date. We also included any cancer diagnoses made in the census stay.

The source of all data related to hospital stays was the Scottish Morbidity Record 01 (SMR01) (13). The measure of deprivation used was the Scottish Index of Multiple Deprivation (SIMD) (14). This is an area based deprivation score, which ranks areas according to a relative measure of deprivation where SIMD 1 represents the 20% most deprived areas in Scotland and SIMD 5 represents the 20% least deprived areas. Linkage to National Records of Scotland (NRS) death records (15) allowed us to follow up patients for a year and match time of death to the admission record (16). We limited the analysis to Scottish residents and excluded records where record linkage was not possible due to omissions or errors. We calculated mortality rates for the general Scottish population in 2015 from NRS death records and the NRS mid-year population estimates for 2015.

Statistical Analysis

We provided statistical summaries in relation to potential risk factors for numbers of admissions and total deaths in the follow-up year, including mortality at seven days, thirty days, three months, six months, nine months and twelve months from the census date. Risk factors were patient demographics (age, sex and deprivation), number of emergency admissions in the year

For peer review only - http://bmjopen.bmj.com/site/about/guidelines.xhtml

prior to the census admissions, number of bed-days in the previous year, having a cancer diagnosis (see Methods for definition) and primary diagnosis in the census admission. We classified primary diagnoses at census admission and primary causes of deaths using the NRS classification for causes of death in Scotland (17) and documented whether death occurred in a hospital, a care home or other institution, or at a private address.

We produced Kaplan-Meier plots for each risk factor (age, sex, cancer diagnosis, and deprivation) to examine differences in survival between groups of patients. We modelled survival in days using multivariate Cox proportional hazards models using R 3.3.2. Follow-up was 366 days as 2016 was a leap year. We censored patients surviving beyond 366 days from the date of their census emergency admission. We conducted univariate analysis to examine the hazard ratio associated with the individual variables. Sex, age, deprivation and cancer diagnosis were all included in the multivariate Cox regression to determine whether these factors were independent predictors of survival.

There was some evidence of non-proportionality in the Schoenfield residuals plot for cancer diagnosis, (although the hazard ratio was always greater than one and quantitative test for a linear trend was non-significant). To test the robustness of our model to this slight non-proportionality and to further investigate differences between cancer and non-cancer patients, we repeated the multivariate Cox regression analysis for cancer and non-cancer patients separately.

Results

We identified 10,477 patients with emergency admissions to medicine during the two week period 18th to 31st March 2015. There were more females (52.1%) than males (47.9%). Most patients were 60 years or older (62.6%), and 14.0% were 85 or older. A greater proportion of admissions came from the most deprived areas (SIMD 1, 29.5%) compared to the least deprived areas (SIMD 5, 13.3%). A total of 1,565 (14.9%) of patients had been given a cancer diagnosis in the previous five years (Table 1).

For peer review only

Table 1: Characteristics of patient cohort and mortality rates

Age group	No. of Admissions	%	Deaths within 7 days	%	Deaths within 30 days	%	Deaths within 3 months	%	Deaths within 6 months	%	Deaths within 9 months	%	Deaths within 1 year	%
0-59	3,915	37.4%	27	0.7%	80	2.0%	179	4.6%	227	5.8%	277	7.1%	309	7.9%
60-64	710	6.8%	12	1.7%	35	4.9%	79	11.1%	104	14.6%	125	17.6%	132	18.6%
65-69	926	8.8%	27	2.9%	64	6.9%	133	14.4%	172	18.6%	207	22.4%	234	25.3%
70-74	1,066	10.2%	39	3.7%	98	9.2%	192	18.0%	229	21.5%	270	25.3%	297	27.9%
75-79	1,175	11.2%	27	2.3%	89	7.6%	203	17.3%	263	22.4%	315	26.8%	351	29.9%
80-84	1,213	11.6%	53	4.4%	125	10.3%	243	20.0%	320	26.4%	376	31.0%	406	33.5%
85+	1,472	14.0%	88	6.0%	202	13.7%	369	25.1%	465	31.6%	560	38.0%	617	41.9%
**Sex**														
Female	5,463	52.1%	145	2.7%	332	6.1%	666	12.3%	857	15.7%	1,031	18.9%	1,138	20.8%
Male	5,014	47.9%	128	2.6%	361	7.2%	732	14.6%	923	18.4%	1,099	21.9%	1,208	24.1%
**SIMD**														
SIMD1 (most deprived)	3,092	29.5%	78	2.5%	204	6.6%	372	12.0%	473	15.3%	564	18.2%	621	20.1%
SIMD2	2,478	23.7%	66	2.7%	155	6.3%	342	13.8%	436	17.6%	519	20.9%	574	23.2%
SIMD3	1,844	17.6%	47	2.5%	119	6.5%	255	13.8%	309	16.8%	374	20.3%	411	22.3%
SIMD4	1,667	15.9%	43	2.6%	121	7.3%	236	14.2%	305	18.3%	370	22.2%	397	23.8%
SIMD5 (least deprived)	1,396	13.3%	39	2.8%	94	6.7%	193	13.8%	257	18.4%	303	21.7%	343	24.6%
**Cancer diagnosis**														
No	8,912	85.1%	187	2.1%	414	4.6%	799	9.0%	1,055	11.8%	1,318	14.8%	1,476	16.6%
Yes	1,565	14.9%	86	5.5%	279	17.8%	599	38.3%	725	46.3%	812	51.9%	870	55.6%
**Total**	**10,477**	100.0%	**273**	2.6%	**693**	6.6%	**1,398**	13.3%	**1,780**	17.0%	**2,130**	20.3%	**2,346**	**22.4%**

For peer review only

Deaths following the census admission

There were 2,346 deaths (22.4% mortality) in the year following the census admission. Table 1 shows the number and percentage of deaths that occurred at different time intervals during the year. Six hundred and ten patients died during the census admission (5.8% of all admissions and 26% of all deaths) while 1,736 of the deaths that occurred during the year did so after the patient had been discharged (74% of all deaths). Overall, men were more likely to die than women (24.1% vs. 20.8%) and this higher mortality was demonstrated within each age group. (Table 2 and Figure 1). Mortality rose steeply with age and was five times higher at one year for patients aged 85 years and over compared to those who were under 60 years of age (41.9% vs. 7.9%). A slightly lower proportion of patients from the most deprived areas (SIMD 1) died during follow-up (20.1%) compared to the less deprived quintiles (ranging from 22.3-24.6%). Cancer patients had a much higher mortality rate (55.6% mortality at one year) than patients without a cancer diagnosis (16.6%) (Table 1).

Cause of death and place of death

Three categories of primary cause of death accounted for almost three quarters of all deaths in the cohort. These were malignant neoplasms (33.8%, with the most common subgroup being cancer of trachea, bronchus and lung); circulatory diseases (22.5% mainly ischaemic heart disease); and respiratory disease (17.9%, mainly chronic lower respiratory tract disease). The most common place of death was an NHS hospital which accounted for 1,594 (67.9%) of the 2,346 deaths. The remainder of the deaths occurred either at home or other private address (17.8%); or in a care home or other institution (14.3%).

Previous admissions

For peer review only

Overall, 48.4% of patients had been admitted as an emergency to hospital in the year before the census admission and 51.9% of survivors of the census admission had at least one emergency hospital admission during follow up. There was a linear relation between numbers of bed days occupied in the year before the census admission and mortality. Those who had no bed days in the previous year had only 14.2% mortality in the year following their census admission compared to 43.9% mortality for those with over 30 bed-days in the previous year (Table 3) (Chi squared = 624.6, df = 5, $p < 0.001$). By contrast the relation between number of emergency

For peer review only

Table 2: Cox regression analysis for mortality in one year of follow-up, for all patients in the cohort

	No. of deaths	No. of patients	Unadjusted Hazard ratio (95% CI)	p-value	Adjusted Hazard ratio (95% CI)	p-value
**Age**						
<60	309	3,915	1	-	1	-
60-64	132	710	2.51 (2.05-3.08)	<0.001	2.17 (1.77-2.66)	<0.001
65-69	234	926	3.53 (2.98-4.18)	<0.001	2.74 (2.31-3.26)	<0.001
70-74	297	1,066	4.01 (3.42-4.7)	<0.001	3.08 (2.62-3.61)	<0.001
75-79	351	1,175	4.28 (3.68-4.99)	<0.001	3.32 (2.84-3.87)	<0.001
80-84	406	1,213	4.99 (4.31-5.79)	<0.001	4.36 (3.75-5.06)	<0.001
85+	617	1,472	6.60 (5.76-7.57)	<0.001	6.07 (5.29-6.97)	<0.001
**Sex**						
Females	1,138	5,463	1	-	1	-
Males	1,208	5,014	1.18 (1.09-1.28)	<0.001	1.23 (1.13-1.33)	<0.001
**Deprivation**						
SIMD 5 = least deprived	343	1,396	1	-	1	-
SIMD 4	397	1,667	0.97 (0.84-1.12)	0.678	1.06 (0.91-1.22)	0.461
SIMD 3	411	1,844	0.90 (0.78-1.04)	0.146	1.04 (0.9-1.20)	0.577
SIMD 2	574	2,478	0.94 (0.82-1.07)	0.342	1.12 (0.98-1.28)	0.099
SIMD 1= most deprived	621	3,092	0.80 (0.7-0.91)	<0.001	1.13 (0.99-1.29)	0.073
**Cancer diagnosis**						
No	1,476	8,912	1	-	1	-
Yes	870	1,565	4.53 (4.16-4.92)	<0.001	3.79 (3.48-4.13)	< 0.001

For peer review only

admissions in the preceding year and mortality, although significant, was non-linear (Chi-squared = 371.7, df = 5, p <0.001). Patients with no emergency admissions in the year prior to their census admission had 15.1% mortality in the follow-up year, those with four or five emergency admissions in the previous year had 35.2% mortality at one year, but patients with more than eleven had lower mortality (18.0%) (Table 3).

Table 3: Number of admissions and % mortality by bed-days in hospital and number of emergency admissions in the year preceding the census admission

Bed days	No. of admissions (% of all admissions)	No. of deaths (% mortality)
0	5,345 (51.0)	759 (14.2)
1-5	1,667 (15.9)	355 (21.3)
6-10	880 (8.4)	269 (30.6)
11-15	533 (5.1)	164 (30.8)
16 - 30	886 (8.5)	287 (32.4)
31+	1,166 (11.1)	512 (43.9)
**Emergency admissions**		
**0**	5,410 (51.6)	819 (15.1)
**1**	2,296 (21.9)	629 (27.4)
**2 - 3**	1,810 (17.3)	594 (32.8)
**4 - 5**	543 (5.2)	191 (35.2)
**6 - 10**	329 (3.1)	97 (29.5)
**11+**	89 (0.8)	16 (18.0)
**Total**	**10,477 (100.0)**	**2,346 (22.4)**

Comparison with the general population

We calculated age standardized mortality rates using the 2013 European Standard Population to take account of the differences in the age distribution of our emergency medical admissions and the Scottish population. Age standardised mortality for men was 122.0 per 1,000 (95% CI 113.0 to 131.0) which was 9 times higher than that of the Scottish population (13.4 per 1,000, 95% CI 13.3 to 13.6) from which they were derived. Age standardised mortality rates for women in our cohort and the general population were 98.9 per 1,000 (90.9 to 106.9) and 10.0 (95% CI 9.8 to 10.1) respectively, indicating a 10 fold increase in risk. Results for both sexes combined were 110.4 (95% CI 104.4 to 116.5) for the cohort and 11.7 (95% CI 11.6 to 11.8) for the Scottish population, a 9 fold increase in risk.

For peer review only

Cox regression for all patients in the cohort

Gender, age, deprivation and cancer diagnosis all predicted mortality in the univariate analysis (Table 2, Figure 2) but in the multivariate analysis only gender, age and cancer were independent predictors of death (Table 2). Men were 1.23 (CI 1.13-1.33) times more likely to die than women after adjusting for other risk factors. Older patients had an increased risk of death: the adjusted hazard ratio for those aged over 85 compared to those aged under 60 was 6.07 (CI 5.29-6.97). Cancer diagnosis was also an important independent predictor of death, with nearly four times increase in risk (HR 3.79, CI 3.48-4.13). By contrast deprivation did not predict outcome once other factors were taken into account.

For peer review only

Cox regression for cancer and non-cancer patients separately

Men were more likely to die than women in both cancer and non-cancer patient groups. The effect size was similar with adjusted hazards ratios for men compared to women of 1.23 (CI 1.08-1.41) and 1.30 (CI 1.17-1.44) in cancer and non-cancer patients respectively. Absolute mortality increased with age in both groups but was always higher in patients with a cancer diagnosis (Table 4). Compared to those aged under 60 years non-cancer patients over 85 had a higher adjusted hazard ratio of 10.95 (CI 9.17-13.07) than cancer patients of the same age (adjusted hazard ratio 1.58 with CI 1.24-2.00). This was a consequence of lower absolute mortality in younger patients who did not have cancer (47.0 per 1000 for non-cancer patients under 60 vs. 463.3 per 1000 for cancer patients under 60). Amongst non-cancer patients the most deprived quintile (SIMD 1) had a slightly higher adjusted hazard ratio than the least deprived quintile (SIMD 5), although the effect was small and the statistical significance was marginal (HR 1.21, CI 1.02-1.43, p=0.029) (Table 4). No significant effect of deprivation was seen in cancer patients.

For peer review only

Table 4: Cox regression analysis for mortality at one year in cancer and non cancer patients

	Cancer patients					Non-cancer patients				
	No. of deaths	No. of patients	Crude mortality rate (per 1,000)	Adjusted hazard ratio (95%CI)	p-value	No. of deaths	No. of patients	Crude mortality rate (per 1,000)	Adjusted hazard ratio (95%CI)	p-value
**Age**										
<60	139	300	463	1	-	170	3,615	47	1	-
60-64	64	118	542	1.27 (0.95-1.71)	0.109	68	592	115	2.52 (1.91-3.34)	<0.001
65-69	124	212	585	1.36 (1.06-1.73)	0.014	110	714	154	3.48 (2.74-4.43)	<0.001
70-74	147	258	570	1.37 (1.08-1.72)	0.008	150	808	186	4.35 (3.49-5.42)	<0.001
75-79	139	264	527	1.19 (0.94-1.5)	0.150	212	911	233	5.58 (4.56-6.83)	<0.001
80-84	125	200	625	1.53 (1.2-1.95)	<0.001	281	1,013	277	7.09 (5.86-8.59)	<0.001
85+	132	213	620	1.58 (1.24-2)	<0.001	485	1,259	385	10.95 (9.17-13.07)	<0.001
**Sex**										
Females	381	742	513	1	-	757	4,721	160	1	-
Males	489	823	594	1.23 (1.08-1.41)	0.002	719	4,191	172	1.3 (1.17-1.44)	<0.001
**Deprivation**										
SIMD 5 = least deprived	140	255	549	1	-	203	1,141	178	1	-
SIMD 4	175	293	597	1.18 (0.95-1.48)	0.138	222	1,374	162	0.99 (0.82-1.2)	0.898
SIMD 3	151	269	561	1.08 (0.86-1.36)	0.503	260	1,575	165	1.02 (0.85-1.22)	0.843
SIMD 2	206	380	542	1.04 (0.83-1.29)	0.745	368	2,098	175	1.15 (0.97-1.36)	0.117
SIMD 1 = most deprived	198	368	538	1.05 (0.84-1.3)	0.669	423	2,724	155	1.21 (1.02-1.43)	0.029

For peer review only

Discussion

We confirm previous findings in an incident rather than prevalent population of emergency medical admissions to Scottish hospitals in 2015. Over 1 in 5 patients died within a year of their census admission with three quarters of the deaths occurring following rather than during that admission. Mortality rose steeply with age and was five times higher at one year for patients aged 85 years and over compared to those who were under 60. Our new findings are that likelihood of death was more closely related to age and to a cancer diagnosis than it was to gender or social deprivation. Over half of all cancer patients died during the 12 months of follow up. Cancer patients were more than three times likely to die than patients without a cancer diagnosis. Mortality was linearly related to the number of bed days occupied but not to the number of admissions in the year before the census admission. Age-sex standardized mortality for men and women was nine and ten times higher respectively than the general population from which they were derived.

These findings have important implications for health and social care. Around 550,000 people die in the UK each year. This number is expected to rise to 615,000 deaths per year by 2030 (18). These deaths commonly occur in hospital and are frequently preceded by one or more emergency hospital admissions. There were over 6 million emergency admissions to NHS hospitals in England (19) and Scotland (20) in 2015-16. Emergency admission to hospital therefore provides an unparalleled opportunity to initiate discussions on end of life care if appropriate and if such discussions have not already begun.

The continuing rise in emergency admissions to hospital (19) likely reflects an increase in life expectancy that is not always healthy life expectancy. The Office for National Statistics has estimated that between 2013 and 2015 UK men at age 65 could expect to live for a further 18.5 years with 10.3 of these years in good health. The corresponding figures for women aged 65 are 20.9 and 11.1 years respectively. Thus men and women aged 65 can expect to live just over half of their remaining years in good health (21). Similar findings have been reported by European (22) and US investigators (23). The unintended consequence of efforts to prevent heart disease and stroke in middle age may be an increase in comorbidity and ill health in later life (24).

The General Medical Council considers that patients are approaching the end of life when they are likely to die within 12 months. This definition of end of life includes patients whose death is imminent, those with advanced progressive incurable conditions and patients with general frailty and coexisting conditions that mean they are expected to die within 12 months (5). The Gold

For peer review only - http://bmjopen.bmj.com/site/about/guidelines.xhtml

Standards Framework Proactive Identification Guidance and the Supportive and Palliative Care Indicators Tool may be used to identify people in the latter two groups whose health is deteriorating and who may be entering the last year of life. Both suggest asking the Surprise Question ("Would you be surprised if the patient were to die in the next year, months, weeks or days?") and looking for specific clinical indicators of decline relating to the three broad trajectories of illness: cancer, organ failure and frailty (3,25).

There is now a continuum of interventions possible within the scope of modern medicine. While active treatment and full escalation to critical care settings are indicated for many patients this may not always be in the best interests of the frail older adult, for whom a holistic end of life approach may be more appropriate. We know that this is often not addressed during emergency medical admission and that doctors do not always feel comfortable initiating end of life discussions with patients and their families. This is a universal challenge for clinicians caring for cancer patients (10,26), patients with organ failure (27,28), and patients with frailty and dementia (29-31); also for clinicians working in the UK (27,30,32-34), Europe (31,35), North America (10,26,28,29) and Australasia (36).

Anticipatory or advance care planning is now widely recommended (3-7). It is disappointing therefore that up to 26% cancer patients, 59% of those with organ failure and 34% with frailty do not have anticipatory care plans in place before death (34). This means that a request to ask 'difficult questions' relating to cardiopulmonary resuscitation and escalation to high dependency or intensive care when a patient is admitted as an emergency to medicine can sometimes cause unintended distress (37). All clinicians involved in caring for patients at the end of life have a responsibility to communicate effectively with patients, their families and members of the multi-disciplinary team in order to explore treatment goals and make key decisions (38,39). Good anticipatory care planning in hospital (40) could mean that the next time a frail older patient becomes unwell at home a decision could be made to nurse him or her at home with support from community palliative care services rather than admit to hospital, thereby avoiding the indignity of treatment that prolongs their suffering.

In conclusion, we believe these data may help identify patients admitted to hospital as medical emergencies who are at greatest risk of dying not only during admission but also in the following 12 months. We would like to think our results will give doctors the confidence to initiate end of life care discussions when this might be more appropriate than active treatment or escalation to critical care settings. Ultimately we believe it is wrong to deny the need for an

approach at the end of life that might provide care that is more humane and perhaps less costly (41,42).

Word count 3079 excl abstract

For peer review only

Copyright

The corresponding author has the right to grant on behalf of all authors and does grant on behalf of all authors, a worldwide licence to the Publishers and its licensees in perpetuity, in all forms, formats and media (whether known now or created in the future), to i) publish, reproduce, distribute, display and store the Contribution, ii) translate the Contribution into other languages, create adaptations, reprints, include within collections and create summaries, extracts and/or, abstracts of the Contribution, iii) create any other derivative work(s) based on the Contribution, iv) to exploit all subsidiary rights in the Contribution, v) the inclusion of electronic links from the Contribution to third party material where-ever it may be located; and, vi) licence any third party to do any or all of the above.

Contributorship

Chris Isles, Emily Moore, Lauren Schofield, Rosalia Munoz-Arroyo and David Clark designed the study. Emily Moore was responsible for record linkage and statistical analyses. Chris Isles wrote the first draft, Alice Radley produced the second draft and all authors contributed to the final draft.

Funding statement

This research received no specific grant from any funding agency in the public, commercial or not-for-profit sectors. Professor David Clark is supported by a Wellcome Trust Investigator Award, Grant Number103319/Z/13/Z.

Ethical approval

Not required

Competing interests

All authors have completed the ICMJE uniform disclosure form at www.icmje.org/coi_disclosure.pdf and declare: no support from any organisation for the submitted work; no financial relationships with any organisations that might have an interest in the submitted work in the three previous years; no other relationships or activities that could appear to have influenced the submitted work

Transparency declaration

The lead author affirms that the manuscript is an honest, accurate, and transparent account of the study being reported; that no important aspects of the study have been omitted; and that any discrepancies from the study as planned (and, if relevant, registered) have been explained.

Data sharing

The lead author on behalf of co-authors is happy to grant access to all study data on request.

For peer review only

References

1. Clark D, Armstrong M, Allan A, Graham F, Carnon A, Isles C.. Imminence of death among hospital inpatients: prevalent cohort study. *Palliative Medicine* 2014; 28: 474-479.

2. Clark D, Schofield L, Graham F, Gott M, Jarlbaek L, Likelihood of death within one year of a national cohort of hospital inpatients in Scotland. *Journal of Pain and Symptom Management* Accessed at https://dx.doi.org/10.1016/j.jpainsymman.2016.05.07 on 10th October 2017

3. The Gold Standards Framework Proactive Identification Guidance, 2016. Accessed at www.goldstandardsframework.org.uk/PIG 10th September 2017

4. NICE Quality Standard 13. End of life care for adults. First published 28th November 2011. Accessed at www.nice.org.uk/guidance/qs13, 10th September 2017

5. General Medical Council. Treatment and care towards the end of life: good practice in decision making. First published 20th May 2010. Accessed at www.gmc-uk.org/guidance/ethical_guidance/end_of_life_care 10th September 2017

6. NHS England. Actions for end of life care: 2014-2016. First published 11th November 2014. Accessed at www.england.nhs.uk/actions-eolc on 10th September 2017

7. Health Improvement Scotland. Palliative and end of life care. First published March 2013. Accessed at www.healthcareimprovementscotland.org on 10th September 2017

8. Mort D, Lansdown M, Smith N, Protopapa K, Mason M. For better or worse? A report by the National Confidential Enquiry into Patient Outcome and Death, 2008. Accessed at https://www.ncepod.org.uk/SACT-report on 4th October 2017

9. Weeks JC, Catalans PJ, Cronin A et al. Patients' expectations about effects of chemotherapy for advanced cancer. *N Engl J Med* 2012; 367: 1616-25

10. Keating NL, Landrum MB, Rogers SO et al. Physician factors associated with discussions about end of life care. *Cancer* 2010; 116: 998-1006

11. World Health Organisation, 2010, International Classification of Diseases, Tenth Revision (ICD-10),

12. ISD Scotland, 2010, Scottish cancer registry, http://www.isdscotland.org/Health-Topics/Cancer/Scottish-Cancer-Registry/

13. ISD Scotland, 2017, General Acute Inpatient and Day Case - Scottish Morbidity Record (SMR01), http://www.ndc.scot.nhs.uk/National-Datasets/data.asp?ID=1&SubID=5

14. APS Group Scotland, 2016. Introducing The Scottish Index of Multiple Deprivation 2016, A National Statistics publication for Scotland. Produced for The Scottish Government by APS Group Scotland. Accessed at http://www.gov.scot/Resource/0050/00504809.pdf.

15. National Records of Scotland, 2017. Vital events: Deaths. Accessed at http://www.nrscotland.gov.uk/statisitics-and-data/statistics/statistics-by-theme/vital-events/deaths

16. National Records of Scotland, 2016a. Statistics by theme. Accessed at http://www.nrscotland.gov.uk/statistics-and-data/statistics/statistics-by-theme.

17. National Records of Scotland, 2016b. Vital Events Reference Tables 2015, Section 6: Deaths – Causes. Accessed at https://www.nrscotland.gov.uk/statistics-and-data/statistics/statistics-by-theme/vital-events/general-publications/vital-events-reference-tables/2015/section-6-deaths-causes

18. Office for National Statistics. Underlying death data from the National Population Projection Accuracy Report, released July 2015. Accessed at https://www.ons.gov.uk/peoplepopulationandcommunity/birthsdeathsandmarriages/deaths/adhocs/004886underlyingdeathsdatafromthenationalpopulationprojectionsaccuracyreportreleasedjuly2015 on 7th October 2017

19. NHS Digital. Hospital admitted patient care activity. England, 2015-16. First published 9th November 2016. Accessed at http://www.content.digital.nhs.uk/catalogue/PUB22378 on 11th September 2017

20. ISD Scotland. Inpatient and day case activity: Emergency admissions by NHS Board and Health and Social Care partnership, October 2016. Accessed at https://www.isdscotland.org/health-topics/hospital-care/inpatient-and-day-case-activity on 7th October 2017

21. Office for National Statistics. Health state life expectancies UK: 2013-2015. First published 29th November 2016. Accessed at

For peer review only

https://www.ons.gov.uk/releases/healthstatelifeexpectanciesuk2013to2015 on 11th September 2017

22. Eurostat Statistics Explained. Healthy Life Years Statistics. Accessed at http://ec.europa.eu/eurostat/statistics-explained/index.php/healthy-life-years-statistics.

23. Centers for Disease Control and Prevention. State specific healthy life expectancy at age 65 years – United States, 2007-2009. MMWR 2013; 62: 561-566

24. Srivastava D. Is prevention better than cure? Research note for the European Commission, 2008. Accessed at https://www.ec.europa.eu/social/BlobServlet?docId=3792&langId=en on 7th October 2017

25. NHS Lothian. Supportive and Palliative care Indicators Tool (SPICT), last updated 18th April 2017 Accessed at https://www.spict.org on 10th October 2017

26. Kumar P, Temel JS. End of life discussions in patients with advanced cancer. *J Clin Oncology* 2013; 31: 3315-19

27. Barclay S, Momen N, Case-Upton S, Kuhn I, Smith E. End of life care conversations with heart failure patients: a systematic literature review and narrative synthesis. *Br J General Practice* 2011; 61: e49-e62.

28. Ahluwalia SC, Levin JR, Lorenz KA, Gordon HS. "There's no cure for this condition": how physicians discuss advance care planning in heart failure. *Patient Educ Counselling* 2013; 91: 200-5

29. Koller K, Rockwood K. Frailty in older adults: implications for end of life care. *Cleveland Clinic Journal of Medicine* 2013; 80:168-74.

30. Eynon T, Lakhani MK, Baker R. Never the right time: advance care planning with frail and older people. *Br J General Practice* 2013; 63: 511-2

31. Geiger K, Schneider N, Bleidorn J, Klindtworth K, Junger S, Muller-Mundt G. Caring for frail older people in the last year of life - the general practitioner's view. *BMC Palliative Care* 2016; 15: 52-61

32. Commission for Healthcare, Audit and Inspection. A report on second-stage complaints about the NHS in England, April 2008. ISBN: 978-1-84562-180-3

For peer review only

33. Sleeman KE. End of life communication: let's talk about death. *J Roy Coll Phys Edinburgh* 2013; 43: 197-9

34. Tapsfield J, Hall C, Lunan C, McCutcheon H, McLoughlin P, Rhee J et al. Many people in Scotland now benefit from anticipatory Care before they die: an after death analysis and interviews with general practitioners. *BMJ Supportive and Palliative Care* 2016. Accessed online doi:10.1136/bmjspcare-2015-001014

35. De Vlaminck A, Pardon K, Beernaert et al. Barriers to advance care planning in cancer, heart failure and dementia patients: a focus group study on general practitioners' views and experiences. *PLOS One* 2014; 9: e84905

36. Gott M, Frey R, Raphael D, O'Callaghan A, Robinson J, Boyd M. Palliative care need and management in the acute hospital setting: a census of one New Zealand hospital. *BMC Palliative Care* 2013; 12: 15

37. The Queen on the Application of David Tracey v Cambridge University Hospitals NHS Foundation Trust, 2014. Neutral Citation Number: (2014) EWCA Civ 822. Accessed at www.judiciary.gov.uk/2014/06 on 28th October 2017

38. Finlay S, Wilson M, Isles C. Assessment of frail older adults and end of life. *GM* 2016; 12: 29-33

39. Fritz Z, Slowther A-M, Perkins GD. Resuscitation policy should focus on the patient not the decision. *BMJ* 2017; 356: j813

40. Radley A, Osborne H, McKenzie T, Lightbody C. Do hospital anticipatory care plans improve patient care? An evaluation of end of life care in a District general hospital. Poster presented at Scottish Partnership for Palliative Care, RCPE 2017 Accessed at https://www.palliativecarescotland.org.uk/content/annual-conference-2017/ on 9th October 2017

41. Scitovsky A. The high cost of dying: what do the data show? *Millbank Q* 2005; 83: 825-41

42. Gawande A, 2008. Letting go: what should Medicine do when it can't save your life? Accessed at https://www.newyorker.com/magazine/2010/08/02/letting-go-2 on 8th October 2017

For peer review only

For peer review only

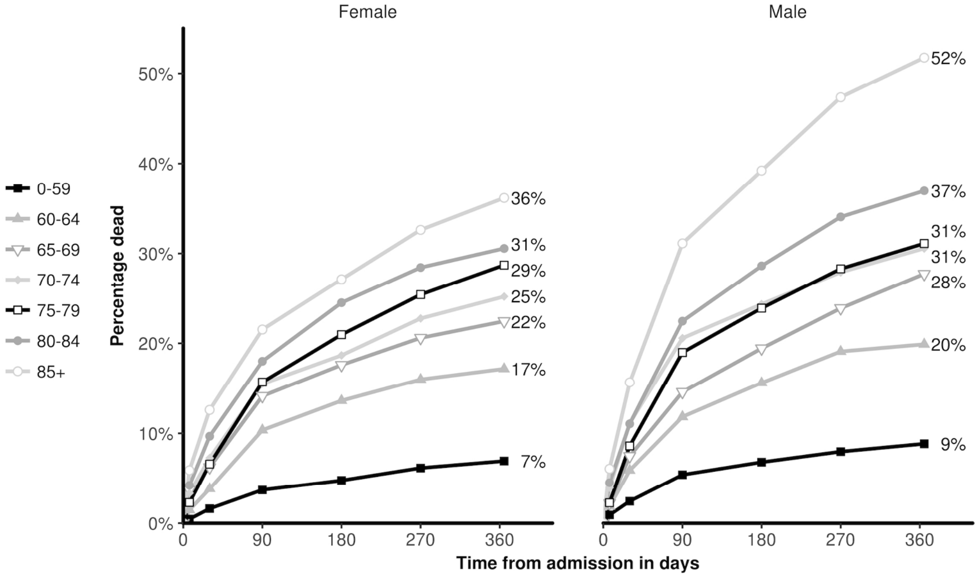

Percentage mortality in females (left panel) and males (right panel) by age and days from admission

199x119mm (300 x 300 DPI)

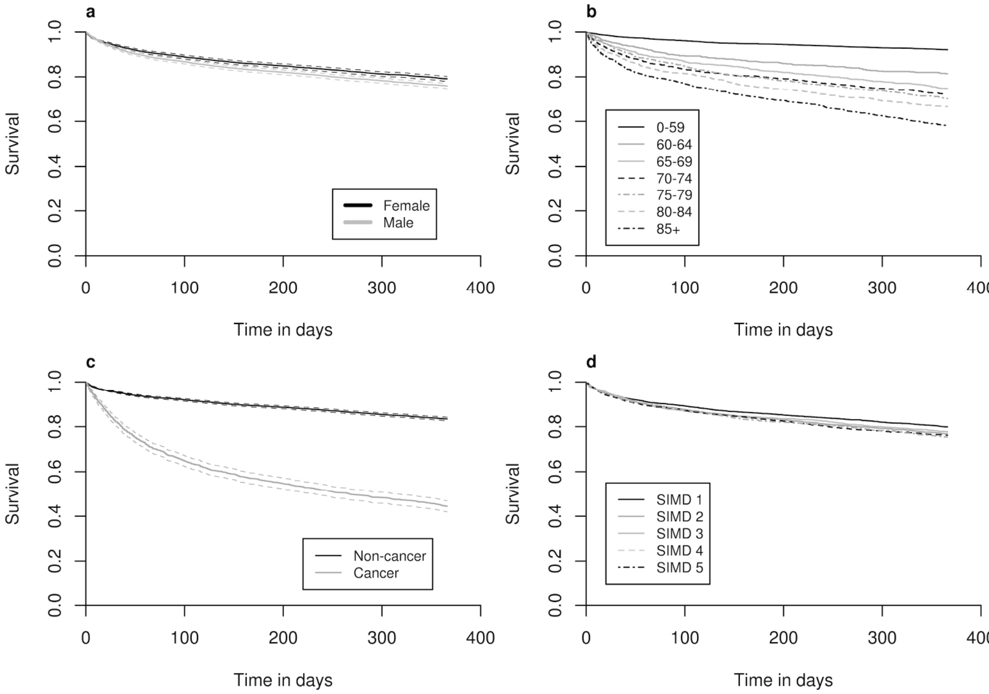

Kaplan-Meier plots showing survival by gender (panel a), age (panel b), cancer diagnosis (panel c) and deprivation category (panel d)

199x140mm (300 x 300 DPI)

Original Article

PALLIATIVE MEDICINE

Palliative Medicine
1–6
© The Author(s) 2014
Reprints and permissions:
sagepub.co.uk/journalsPermissions.nav
DOI: 10.1177/0269216314526443
pmj.sagepub.com
SAGE

Imminence of death among hospital inpatients: Prevalent cohort study

David Clark[1], Matthew Armstrong[2], Ananda Allan[3], Fiona Graham[4], Andrew Carnon[3] and Christopher Isles[5]

Abstract

Background: There is a dearth of evidence on the proportion of the hospital population at any one time, that is in the last year of life, and therefore on how hospital policies and services can be oriented to their needs.

Aim: To establish the likelihood of death within 12 months of a cohort of hospital inpatients on a given census date.

Design: Prevalent cohort study.

Participants: In total, 10,743 inpatients in 25 Scottish teaching and general hospitals on 31 March 2010.

Results: In all, 3098 (28.8%) patients died during follow-up: 2.9% by 7 days, 8.9% by 30 days, 16.0% by 3 months, 21.2% by 6 months, 25.5% by 9 months and 28.8% by 12 months. Deaths during the index admission accounted for 32.3% of all deaths during the follow-up year. Mortality rose steeply with age and was three times higher at 1 year for patients aged 85 years and over compared to those who were under 60 years (45.6% vs 13.1%; $p < 0.001$). In multivariate analyses, men were more likely to die than women (odds ratio: 1.18, 95% confidence interval: 0.95–1.47) as were older patients (odds ratio: 4.99, 95% confidence interval: 3.94–6.33 for those who were 85 years and over compared to those who were under 60 years), deprived patients (odds ratio: 1.17, 95% confidence interval: 1.01–1.35 for most deprived compared to least deprived quintile) and those admitted to a medical specialty (odds ratio: 3.13, 95% confidence interval: 2.48–4.00 compared to surgical patients).

Conclusion: Large numbers of hospital inpatients have entered the last year of their lives. Such data could assist in advocacy for these patients and should influence end-of-life care strategies in hospital.

Keywords

Inpatients, hospitalisation, palliative care, terminal care, end-of-life care, social deprivation, mortality

What is already known about this topic?

- Understanding of the high proportion of deaths occurring in hospital is well established.
- Some data exist on the proportion of patients in hospital likely to benefit from palliative care.
- There is evidence of difficulty in making the transition to palliative care for patients in hospital.

What this study adds?

- This is the first study of its kind to establish the proportion of hospital inpatients who die over a period of 12 months from a given date.
- The study shows how the likelihood of death in 12 months is related to male gender, age, admission to a medical specialty and social deprivation.

Implications for practice, theory or policy

- In order that appropriate care plans can be made and delivered for patients, there is a strong need for hospitals to adopt a more vigorous approach to identify patients who are entering the last year of their lives.
- We contend that the culture and organisation of hospitals need to become more attuned to the high proportion of inpatients in imminent need of end-of-life care.

[1]School of Interdisciplinary Studies, University of Glasgow, Dumfries, UK
[2]Healthcare Information Group, Information Services Division, NHS National Services Scotland, Edinburgh, UK
[3]Department of Public Health, NHS Dumfries & Galloway, Dumfries, UK
[4]Glenkens Medical Practice, New Galloway, UK
[5]Dumfries and Galloway Royal Infirmary, NHS Dumfries & Galloway, Dumfries, UK

Corresponding author:
David Clark, School of Interdisciplinary Studies, University of Glasgow, Dumfries Campus, Rutherford/McCowan Building, Bankend Road, Dumfries DG1 4ZL, Scotland, UK.
Email: David.Clark.2@glasgow.ac.uk

For peer review only - http://bmjopen.bmj.com/site/about/guidelines.xhtml

Introduction

There is growing interest in the challenge of providing appropriate end-of-life care to an ageing population, as demands on services increase and as expectations of patients and families change.[1,2] The role of the hospital in the delivery and planning of such care is of major significance, partly because so many patients die there, but also because hospital admission provides an opportunity to identify those who may be approaching death.[3,4] The likelihood of dying in hospital varies across countries, but is generally high. One recent study involving 36 national jurisdictions ranked Scotland 12th from the top for the proportion of all deaths occurring in hospital (59%); Japan (78%) had the highest proportion of hospital deaths.[5] A study of six European countries found significant national variation in the proportions of all deaths that occurred in hospital, from 33.9% (Netherlands) to 62.8% (Wales).[6] Older people are those most likely to die in hospital. The European study showed that in Scotland, among those aged 80–84 years, 62.3% of all deaths were in hospital. In England, the greatest proportion of hospital deaths is in the 65–84 years group, at 61% of all deaths for this group.[7]

A 2001 study in one hospital in England found that 23% of the total inpatient population was identified by staff as having palliative care needs and/or being terminally ill, and 11% were considered suitable for referral to a specialist palliative care bed.[8] A more recent study in two English hospitals using case notes to examine for evidence of palliative care need according to Gold Standards Framework (GSF) prognostic indicator criteria and including the views of medical and nursing staff and patients (or consultees) found that 36.0% of patients were identified as having palliative care needs.[9] In an Australian hospital network, 35% of acute inpatients were identified as having palliation as the goal for their long-term care.[10] In a group of 14 Belgian hospitals, 9.4% of inpatients were identified as 'palliative' by the medical and nursing staff.[11]

Such studies are useful in establishing two factors: the propensity to die in hospital and the proportion of patients in hospital at any one time who may have palliative care needs. A third perspective can be contributed by assessing the proportions and characteristics of those in hospital who are nearing the end of life and using that to shape packages of care for those patients. Knowing more about this group would create greater possibilities for advance care planning for groups of patients, even if individual prognostication is problematic.[12,13] In an acute hospital in New Zealand, it was found that 19.8% of inpatients included in a census met at least one of the GSF prognostic indicators, suggesting that they were likely to be in the last year of life;[14] but it is not known if the prognostication proved accurate. We set out to answer two questions in the Scottish context. First, what proportion of inpatients in Scotland's teaching and general hospitals on a given date will die during the index admission and 3 months, 6 months, 9 months and 12 months later? Second, how does the proportion vary by age, gender, specialty and deprivation score?

Participants and setting

We chose those hospitals in Scotland in which most acute clinical activity occurs, the teaching (n = 7) and large general hospitals (n = 18), as agreed by others. We established how many inpatients, excluding geriatric long stay, were in these hospitals on the census date of 31 March 2010. Teaching hospitals accounted for 4829 patients; general hospitals for 5914. A patient was counted as being in hospital overnight on 31 March 2010 if they had a Scottish Morbidity Record Scheme 01 (SMR01) episode where the admission date was 31 March 2010 or earlier and where the discharge date was 1 April 2010 or later. The source of the hospital data was the national SMR01, which records all inpatient and day case discharges from non-obstetric and non-psychiatric specialties in National Health Service (NHS) hospitals in Scotland.

Methods

We provided statistical summaries for all data using numbers and percentages of deaths at 7 and 30 days, 3 months, 6 months, 9 months and 1 year from the census date. We used multivariate logistic regression models, using R 3.0.1, to determine whether there was any association between potential predictor variables and mortality at 1 year, adjusting for the possible confounding effect of age, gender, a measure of deprivation and whether admission was to a medical or surgical specialty. A univariate logistic regression analysis, also using R 3.0.1, was undertaken to examine the relationship between age and mortality. The measure of deprivation used here is the Scottish Index of Multiple Deprivation 2009 (SIMD09), an area-based deprivation score which groups the Scottish population into five equal quintiles, with quintile 1 representing the 20% most deprived areas in Scotland and quintile 5 the least deprived.[15] The National Records of Scotland office provided information on deaths including the date of death.[16]

Results

We identified 10,743 hospital inpatients on the census date. More were women (54.7%) than men (45.3%). Most (64.1%) were aged 65 years or older. A disproportionate number of admissions belonged to the two most deprived quintiles (50.1%), and more patients had been

For peer review only

admitted to a medical (63.1%) than to a surgical specialty (36.8%).

Table 1 shows that 2.9% had died within 7 days of the census date, 8.9% by 30 days, 16.0% by 3 months, 21.2% by 6 months, 25.5% by 9 months and 28.8% by 12 months.

Descriptive analysis showed that men were more likely to die than women for the majority of age groups at each follow-up time interval (Table 1 and Figure 1). The mortality rate rose steeply with age and was three times higher at 1 year for patients aged 85 years and over compared to those who were under 60 years of age (45.6% vs 13.1%; $p < 0.001$) (Table 1).

The univariate logistic regression showed that every 1 year increase in age at the census date was associated with a 1.04 fold increase in the mortality odds.

In multivariate analyses, men were more likely to die than women (odds ratio (OR): 1.18, 95% confidence interval (CI): 0.95–1.47) as were older patients (OR: 4.99, 95% CI: 3.94–6.33 for those who were 85 years and over compared to those who were under 60 years), deprived patients (OR: 1.17, 95% CI: 1.01–1.35 for most deprived compared to least deprived quintile) and those admitted to a medical specialty (OR: 3.13, 95% CI: 2.48–4.00 compared to surgical patients).

Of the patients, 9.3% died during the index admission, and this accounted for 1001 (32.3%) of all 3098 deaths within the 12-month follow-up period. Around 70% of the deaths (2147 of 3098) occurred more than 30 days after the census date. The 2097 patients who survived the index admission but died within 12 months of the census date required a further 4231 hospital stays (subsequent to the index stay) between 31 March 2010 and their respective dates of death.

Discussion

Our study quantifies the large number of hospital patients who are within the last year of life and produces findings relevant to health-care priority setting. The likelihood of death during the 12 months after our census date was more closely related to age and admission to a medical specialty than to male gender and social deprivation. We have also shown that most of the deaths occur after discharge from hospital and not during the index admission.

Despite the difficulty of prognosticating for individual patients, the scale of the issue revealed here requires a response from both policymakers and clinicians. Our findings support the various initiatives currently underway to raise the profile of end-of-life care in the hospital. Best known of these, and currently the subject of much debate, is the Liverpool Care Pathway (LCP);[17] whatever the outcome of decisions about what should replace the LCP,[18–20] the need is clear for some structured approach to the identification and care of patients in hospital in the last days of life – shown in our data to be 9% of the hospital population. In Ireland, a wider 'systems approach' has been developed known as the Hospice Friendly Hospitals Programme, which seeks to promote improved end-of-life care as part of the 'core business' of the acute hospital.[21] Advance Care Planning with hospital patients likewise seeks to promote good end-of-life care at a point where patients become incapable of participating in medical treatment decisions.[22] Such interventions would benefit from more detailed knowledge of the imminence of death in the hospital population, as described here.

A recent study has highlighted the mismatch between current best practice recommendations on transitions to palliative care in acute hospitals and the observed clinical reality.[23] Two key barriers were identified: (1) the internal momentum of the hospital towards cure inhibited clinicians from standing back and thinking about the overall goals that should inform patient care and (2) decision-making was consultant led, with junior members of the team and particularly nursing colleagues much less involved in discussion about the goals of care. Our data help inform how the approach to such issues might be targeted in the first instance.

The current UK General Medical Council guidance on end-of-life care requires doctors to ensure that death becomes an explicit discussion point when patients are likely to die within 12 months and places a strong emphasis on patient choice rather than 'medical paternalism … however benignly intended'.[24] The 2010 document *The Route to Success in End of Life Care – Achieving Quality in Acute Hospitals*, produced by the National End of Life Care Programme for England, makes a particular point of addressing not only a clinical audience but also board members and senior managers, indicating the need for 'a commitment to support and review end of life care services'.[25] This study supports clinicians and managers to give greater priority to the identification of patients at the end of life and to encourage a more proactive response to their needs.

Strengths and limitations of the study

We are not aware of another study of this type, which requires sophisticated techniques of record linkage that connect data from the hospital system with national death registration data. We provide an analysis covering all teaching and general hospitals in Scotland in a method that could be (where facilities and laws allow) replicated elsewhere. We have not yet been able to establish predictors of death within 12 months that relate to clinical or health indicators, such as diagnosis, co-morbidities or previous use of services.

The study is designed as a prevalent cohort study. There are certain implications to this design. One aspect is that patients with longer hospital stays will have a larger chance

For peer review only

Table 1. Number and percentage of patients in teaching and general hospitals in Scotland on 31 March 2010, dying at intervals after census date.

	In hospital on 30 March 2010, *n* (%)	Deaths within 7 days, *n* (%)	Deaths within 30 days, *n* (%)	Deaths within 3 months, *n* (%)	Deaths within 6 months, *n* (%)	Deaths within 9 months, *n* (%)	Deaths within 1 year, *n* (%)	Death after 30 days (%) of all deaths within 1 year
Gender								
Men	4866 (45.3)	147 (3.0)	458 (9.4)	819 (16.8)	1095 (22.5)	1299 (26.7)	1480 (30.4)	69.1
Women	5877 (54.7)	167 (2.8)	493 (8.4)	897 (15.3)	1183 (20.1)	1439 (24.5)	1618 (27.5)	69.5
Age (years)								
Under 60	3008 (28.0)	30 (1.0)	110 (3.7)	207 (6.9)	283 (9.4)	342 (11.4)	394 (13.1)	72.1
60–64	845 (7.9)	18 (2.1)	60 (7.1)	95 (11.2)	134 (15.9)	167 (19.8)	191 (22.6)	68.6
65–69	1021 (9.5)	38 (3.7)	87 (8.5)	155 (15.2)	205 (20.1)	256 (25.1)	299 (29.3)	70.9
70–74	1185 (11.0)	35 (3.0)	105 (8.9)	191 (16.1)	267 (22.5)	314 (26.5)	355 (30.0)	70.4
75–79	1487 (13.8)	43 (2.9)	148 (10.0)	285 (19.2)	370 (24.9)	437 (29.4)	491 (33.0)	69.9
80–84	1430 (13.3)	63 (4.4)	180 (12.6)	319 (22.3)	412 (28.8)	496 (34.7)	562 (39.3)	68.0
85 and over	1767 (16.4)	87 (4.9)	261 (14.8)	464 (26.3)	607 (34.4)	726 (41.1)	806 (45.6)	67.6
Deprivation (SIMD09)								
Q1 (most)	2936 (27.3)	83 (2.8)	265 (9.0)	474 (16.1)	634 (21.6)	768 (26.2)	874 (29.8)	69.7
Q2	2453 (22.8)	79 (3.2)	230 (9.4)	395 (16.1)	526 (21.4)	629 (25.6)	720 (29.4)	68.1
Q3	1988 (18.5)	53 (2.7)	169 (8.5)	317 (15.9)	425 (21.4)	510 (25.7)	570 (28.7)	70.4
Q4	1800 (16.8)	55 (3.1)	161 (8.9)	304 (16.9)	385 (21.4)	456 (25.3)	504 (28.0)	68.1
Q5 (least)	1515 (14.1)	43 (2.8)	124 (8.2)	222 (14.7)	303 (20.0)	370 (24.4)	424 (28.0)	70.8
Specialty								
Medical	6779 (63.1)	253 (3.7)	757 (11.2)	1357 (20.0)	1793 (26.4)	2134 (31.5)	2410 (35.6)	68.6
Surgical	3954 (36.8)	61 (1.5)	194 (4.9)	359 (9.1)	485 (12.3)	603 (15.2)	686 (17.3)	71.7
Outcome of index admission								
Discharged	9742 (90.7)							
Death	1001 (9.3)							
Hospital type								
Teaching (*n* = 7)	4829 (45.0)							
Large general hospital (*n* = 18)	5914 (55.0)							
Total	10,743 (100)	314 (2.9)	951 (8.9)	1716 (16.0)	2278 (21.2)	2738 (25.5)	3098 (28.8)	69.3

SIMD09: Scottish Index of Multiple Deprivation 2009.

For peer review only

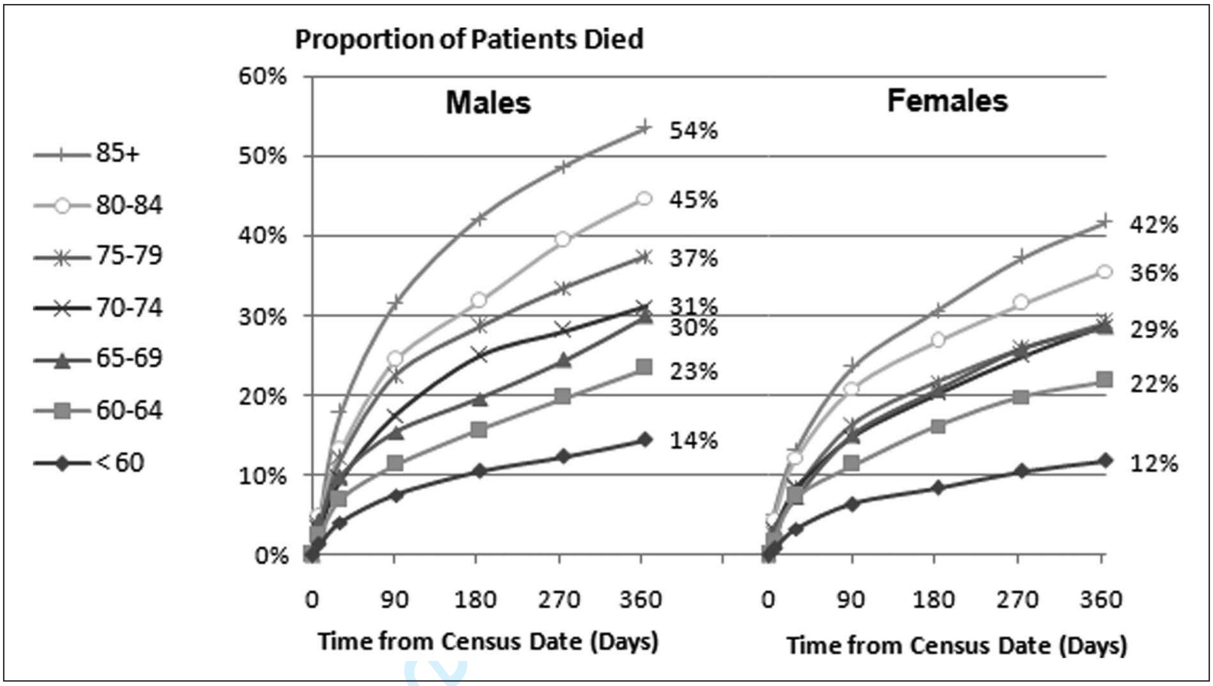

Figure 1. Mortality over time for all patients in teaching and general hospitals in Scotland on 31 March 2010.

of becoming part of the 'sample' (though we would regard ours more properly as a 'population'). Indeed, the probability of being sampled is proportional to length of stay (also known as length-biased sampling). This means that the results are not meaningful for individual patients, though of course that is not the stated interest of this article. But we must acknowledge that our results are likely to be different from a study where sampling was done on day of admission to hospital (an incident sample). Our study is intended to inform system-level debate about care of the hospital population in relation to end-of-life issues. An incident sample would inform thinking about the assessment of patients for end-of-life care needs, as they came into the hospital. Our sampling may also have influenced the relation between deprivation and mortality – with the possibility that more deprived patients are likely to be hospitalised longer.

Conclusion

We have shown in the Scottish context that almost 1 in 10 patients in teaching or general hospitals at any given time will die during that admission. Almost 1 in 3 patients will have died a year later, rising to nearly 1 in 2 for the oldest groups. Hospitals are clearly an important context for end-of-life care, yet there are still difficulties in making the transition to palliative care and in implementing interventions for the imminently dying. In order that appropriate care plans can be made and delivered for patients, there is a strong need for hospitals to adopt a more vigorous approach to identifying patients who are entering the last years of their lives. We contend that the culture and organisation of hospitals need to become more attuned to the high proportion of inpatients in imminent need of end-of-life care.

Acknowledgements

David Clark conceptualised the study, created the research questions, shaped the design and analysis and wrote the first draft of this article; Matthew Armstrong conducted the record linkage and final statistical tests; Ananda Allan shaped the design and analysis and conducted the initial statistical tests; Fiona Graham shaped the design and analysis and contributed to the drafting of this article; Andrew Carnon shaped the design and analysis and contributed to the drafting of this article; Christopher Isles shaped the design and analysis and contributed to the drafting of this article and Eugene Murray (contributor) suggested the idea of tracking mortality in a group of hospital patients. Thanks also to Lindsay Martin and Peter Hutchison.

Declaration of conflicting interests

All authors have completed the ICMJE uniform disclosure form at www.icmje.org/coi_disclosure.pdf and declare that no support was received from any organisation for the submitted work, no financial relationships existed with any organisations that might have an interest in the submitted work in the previous 3 years and no other relationships or activities that could appear to have influenced the submitted work.

Ethical approval

Not required.

For peer review only

Funding

This research received no specific grant from any funding agency in the public, commercial or not-for-profit sectors.

References

1. Gott M and Ingleton C (eds). *Living with ageing and dying: palliative and end of life care for older people*. Oxford: Oxford University Press, 2011.
2. Cohen J and Deliens L (eds). *A public health perspective on end of life care*. Oxford: Oxford University Press, 2012.
3. Al-Qurainy R, Collis E and Feuer D. Dying in an acute hospital setting: the challenges and the solutions. *Int J Clin Pract* 2009; 63(3): 508–515.
4. Toscani F, Di Giulio P, Brunelli C, et al. How people die in hospital general wards: a descriptive study. *J Pain Symptom Manag* 2005; 30: 33–40.
5. Broad JB, Gott M, Kim H, et al. Where do people die? An international comparison of the percentage of deaths occurring in hospital and residential aged care settings in 45 populations, using published and available statistics. *Int J Public Health* 2013; 58(2): 257–267.
6. Cohen J, Houttekier D, Onwuteaka-Philipsen B, et al. Which patients with cancer die at home? A study of six European countries using death certificate data. *J Clin Oncol* 2010; 28(13): 2267–2273.
7. National End of life Care Intelligence Network. Variations in place of death in England inequalities or appropriate consequences of age, gender and cause of death? http://www.endoflifecare-intelligence.org.uk/resources/publications/variations_in_place_of_death (2010).
8. Gott M, Ahmedzai S and Wood C. How many inpatients at an acute hospital have palliative care needs? Comparing the perspectives of medical and nursing staff. *Palliat Med* 2001; 15(6): 451–460.
9. Gardiner C, Gott M, Ingleton C, et al. Extent of palliative care need in the acute hospital setting: a survey of two acute hospitals in the UK. *Palliat Med* 2013; 27(1): 76–83.
10. To T, Greene A, Agar M, et al. A point prevalence survey of hospital inpatients to define the proportion with palliation as the primary goal of care and the need for specialist palliative care. *J Intern Med* 2011; 41(5): 430–433.
11. Desmedt M, Kethulle Y, Deveugele M, et al. Palliative inpatients in general hospitals: a one day observational study in Belgium. *BMC Palliat Care* 2011; 10: 2.
12. Steinhauser KE, Christakis NA, Clipp EC, et al. Factors considered important at the end of life by patients, family, physicians, and other care providers. *JAMA* 2000; 284(19): 2476–2482.
13. Yanneo EG. Determining prognosis and predicting survival in end-of-life care. *Curr Opin Support Palliat Care* 2009; 3(3): 203–206.
14. Gott M, Frey R, Raphael D, et al. Palliative care need and management in the acute hospital setting: a census of one New Zealand hospital. *BMC Palliat Care* 2013; 12: 5.
15. http://www.scotland.gov.uk/Resource/Doc/933/0115249.pdf (accessed 31 January 2014).
16. http://www.gro-scotland.gov.uk/statistics/theme/vital-events/deaths/index.html (accessed 31 January 2014).
17. More care less pathway: a review of the Liverpool Care Pathway Crown Copyright, July 2013, https://www.gov.uk/government/uploads/system/uploads/attachment_data/file/212450/Liverpool_Care_Pathway.pdf (accessed 3 February 2014).
18. Livesley B. The pseudo-Liverpool Care Pathway. *Br J Healthc Manag* 2013; 19(2): 58–59.
19. Torjesen I. Bad press over Liverpool care pathway has scared patients and doctors, say experts. *BMJ* 2013; 346: f175.
20. George R, Martin J and Robinson V. The Liverpool Care Pathway for the dying (LCP): lost in translation and a tale of elephants, men, myopia – and a horse. *Palliat Med* 2014; 28(3): 3–7.
21. Clark D and Graham F. Irish Hospice Friendly Hospitals programme. *BMJ* 2010; 341: c5843.
22. Detering KM, Hancock AD, Reade MC, et al. The impact of advance care planning on end of life care in elderly patients: randomised controlled trial. *BMJ* 2010; 340: c1345.
23. Gott M, Ingleton C, Bennett M, et al. Transitions to palliative care in acute hospitals in England: qualitative study. *BMJ* 2011; 342: d1773.
24. General Medical Council. Treatment and care towards the end of life: good practice in decision-making, www.gmc-uk.org/guidance/ethical_guidance/6858.asp (2010, accessed 24 April 2013).
25. Department of Health. The route to success in end of life care – achieving quality in acute hospitals. *National End of Life Care Programme*, http://www.endoflifecare.nhs.uk/search-resources/resources-search/publications/the-route-to-success-in-end-of-life-care-%e2%80%93-achieving-quality-for-social-work.aspx (2010, accessed 24 April 2013).

For peer review only

STROBE Statement—Checklist of items that should be included in reports of ***cohort studies***

	Item No	Recommendation	Reported on page no
**Title and abstract**	1	(*a*) Indicate the study's design with a commonly used term in the title or the abstract	1
		(*b*) Provide in the abstract an informative and balanced summary of what was done and what was found	2
**Introduction**			
Background/rationale	2	Explain the scientific background and rationale for the investigation being reported	3
Objectives	3	State specific objectives, including any prespecified hypotheses	3
**Methods**			
Study design	4	Present key elements of study design early in the paper	4
Setting	5	Describe the setting, locations, and relevant dates, including periods of recruitment, exposure, follow-up, and data collection	4
Participants	6	(*a*) Give the eligibility criteria, and the sources and methods of selection of participants. Describe methods of follow-up	4
		(*b*) For matched studies, give matching criteria and number of exposed and unexposed	NA
Variables	7	Clearly define all outcomes, exposures, predictors, potential confounders, and effect modifiers. Give diagnostic criteria, if applicable	4,5
Data sources/ measurement	8*	For each variable of interest, give sources of data and details of methods of assessment (measurement). Describe comparability of assessment methods if there is more than one group	4
Bias	9	Describe any efforts to address potential sources of bias	5
Study size	10	Explain how the study size was arrived at	4
Quantitative variables	11	Explain how quantitative variables were handled in the analyses. If applicable, describe which groupings were chosen and why	5
Statistical methods	12	(*a*) Describe all statistical methods, including those used to control for confounding	4,5
		(*b*) Describe any methods used to examine subgroups and interactions	5
		(*c*) Explain how missing data were addressed	3
		(*d*) If applicable, explain how loss to follow-up was addressed	NA
		(*e*) Describe any sensitivity analyses	NA
**Results**			
Participants	13*	(a) Report numbers of individuals at each stage of study—eg numbers potentially eligible, examined for eligibility, confirmed eligible, included in the study, completing follow-up, and analysed	3
		(b) Give reasons for non-participation at each stage	NA
		(c) Consider use of a flow diagram	NA
Descriptive data	14*	(a) Give characteristics of study participants (eg demographic, clinical, social) and information on exposures and potential confounders	5, 6(Table 1), 7, 8
		(b) Indicate number of participants with missing data for each variable of interest	NA
		(c) Summarise follow-up time (eg, average and total amount)	5

For peer review only

Outcome data	15*	Report numbers of outcome events or summary measures over time	6
Main results	16	(*a*) Give unadjusted estimates and, if applicable, confounder-adjusted estimates and their precision (eg, 95% confidence interval). Make clear which confounders were adjusted for and why they were included	9,10
		(*b*) Report category boundaries when continuous variables were categorized	10, 12
		(*c*) If relevant, consider translating estimates of relative risk into absolute risk for a meaningful time period	NA
Other analyses	17	Report other analyses done—eg analyses of subgroups and interactions, and sensitivity analyses	11,12
**Discussion**			
Key results	18	Summarise key results with reference to study objectives	13
Limitations	19	Discuss limitations of the study, taking into account sources of potential bias or imprecision. Discuss both direction and magnitude of any potential bias	14
Interpretation	20	Give a cautious overall interpretation of results considering objectives, limitations, multiplicity of analyses, results from similar studies, and other relevant evidence	13-14
Generalisability	21	Discuss the generalisability (external validity) of the study results	14
**Other information**			
Funding	22	Give the source of funding and the role of the funders for the present study and, if applicable, for the original study on which the present article is based	16

*Give information separately for exposed and unexposed groups.

Note: An Explanation and Elaboration article discusses each checklist item and gives methodological background and published examples of transparent reporting. The STROBE checklist is best used in conjunction with this article (freely available on the Web sites of PLoS Medicine at http://www.plosmedicine.org/, Annals of Internal Medicine at http://www.annals.org/, and Epidemiology at http://www.epidem.com/). Information on the STROBE Initiative is available at http://www.strobe-statement.org.

For peer review only

For peer review only - http://bmjopen.bmj.com/site/about/guidelines.xhtml

BMJ Open

Death within one year among emergency medical admissions to Scottish hospitals: incident cohort study.

Journal:	*BMJ Open*
Manuscript ID	bmjopen-2017-021432.R1
Article Type:	Research
Date Submitted by the Author:	07-Mar-2018
Complete List of Authors:	Moore, Emily ; NHS Scotland National Services Division<br>Munoz-Arroyo, Rosalia; NHS Scotland National Services Division<br>Schofield, Lauren; NHS Scotland National Services Division<br>Radley, Alice ; Dumfries and Galloway Royal Infirmary, Medicine<br>Clark, David; University of Glasgow,, School of Interdisciplinary Studies,<br>Isles, Chris; Dumfries ,
**Primary Subject Heading**:	Palliative care
Secondary Subject Heading:	Emergency medicine, Health policy
Keywords:	emergency medical admissions, mortality, end of life care, anticipatory care plans

SCHOLARONE™
Manuscripts

Death within one year among emergency medical admissions to Scottish hospitals: incident cohort study.

Emily Moore[1], Rosalia Munoz-Arroyo[1], Lauren Schofield[1], Alice Radley[3], David Clark[2] and Chris Isles[3]
[1]NHS National Services Scotland, 1 South Gyle Crescent, Edinburgh EH12 9EB, [2]School of Interdisciplinary Studies, University of Glasgow, and [3]Department of Medicine, Dumfries and Galloway Royal Infirmary, Dumfries DG1 4AP

Emily Moore	Information Analyst
Rosalia Munoz-Arroyo	Principal Information Analyst
Lauren Schofield	Senior Information Analyst
Alice Radley	Core Medical Trainee
David Clark	Professor of Medical Sociology
Chris Isles	Consultant Physician

Correspondence:
Prof Chris Isles
Medical Unit, Dumfries Infirmary,
Dumfries DG2 8RX
Chris.isles@nhs.net
07590 317255

Word count: 3160 excl abstract

Key words: hospital inpatients; emergency medical admissions; mortality; anticipatory care planning; end of life care

For peer review only

For peer review only - http://bmjopen.bmj.com/site/about/guidelines.xhtml

Abstract

Background: It is increasingly recognized that large numbers of hospital inpatients have entered the last year of their lives.

Aim: To establish the likelihood of death within 12 months of admission to hospital; to examine the influence on survival of a cancer diagnosis made within the previous 5 years; and to compare mortality with that of the wider Scottish population.

Design: Incident cohort study.

Setting: 22 hospitals in Scotland.

Participants: This study used routinely collected data from 10,477 inpatients admitted as an emergency to medicine in 22 Scottish hospitals between 18th and 31st March 2015. These data were linked to national death records and the Scottish Cancer Registry.

Primary outcome measures: One year cohort mortality compared to that of the general Scottish population. Patient factors correlating with higher risk of mortality were identified using Cox regression.

Results: There were 2,346 (22.4%) deaths in the year following the census admission. Six hundred and ten patients died during that admission (5.8% of all admissions and 26% of all deaths) while 1,736 died after the census admission (74% of all deaths). Malignant neoplasms (33.8%), circulatory diseases (22.5%) and respiratory disease (17.9%) accounted for almost three quarters of all deaths. Mortality rose steeply with age and was five times higher at one year for patients aged 85 years and over compared to those who were under 60 years of age (41.9% vs. 7.9%) ($p<0.001$). Cancer patients had a higher mortality rate than patients without a cancer diagnosis (55.6% vs. 16.6%) ($p<0.001$). Mortality was linearly related to the number of bed days occupied in the year before the census admission ($p<0.001$) but not to the number of admissions in that year. Age-sex standardized mortality was 110.4 (95% CI 104.4 to 116.5) for the cohort and 11.7 (95% CI 11.6 to 11.8) for the Scottish population, a 9.4 fold increase in risk.

Conclusion: These data may help identify groups of patients admitted to hospital as medical emergencies who are at greatest risk of dying not only during admission but also in the following 12 months.

For peer review only - http://bmjopen.bmj.com/site/about/guidelines.xhtml

Strengths

- This was an incident rather than prevalent cohort study
- The source of all data related to hospital stays was the Scottish Morbidity Record and the Scottish Cancer Registry database.
- Linkage to National Records of Scotland death records allowed us to follow patients for a year and match time of death to the admission record.

Limitations

- If patients emigrated and died abroad during the year of follow up then this would underestimate their mortality. We think this is unlikely to be a source of major imprecision.
- We recognise that our analysis identifies groups of individuals at high risk of death within one year and that it cannot and should not be used to predict an individual's risk of dying.

For peer review only

For peer review only - http://bmjopen.bmj.com/site/about/guidelines.xhtml

Introduction

We have previously shown high 12 month mortality among Scottish hospital inpatients indicating that, for many individuals, admission to hospital is a sentinel event marking the transition to the last year of their lives (1,2). Nearly 1 in 10 patients died during admission, and almost 1 in 3 patients had died by a year later. This increased to nearly 1 in 2 for the over 85 age group. This information highlighted a need for clinicians to alter their approach to patient care in order to identify and address key end of life care needs (1,2). Colleagues in Ireland (3) and New Zealand (4) have conducted similar analyses and drawn similar conclusions. This realistic, patient-focussed approach has been widely advocated by the Gold Standards Framework (5), NICE (6), the General Medical Council (7), NHS England 8) and Health Improvement Scotland (9).

Despite these clear recommendations, many clinicians are reluctant to address end of life issues. A National Confidential Enquiry into Patient Outcome and Death (NCEPOD) review of the care of patients who died within 30 days of receiving systemic anti cancer therapy found that the decision to treat with chemotherapy was inappropriate in 19% cases. This raised questions as to whether cancer patients are given enough information about chemotherapy to enable them to make an informed consent to treatment (10). In a study of patients with metastatic lung and colorectal cancer, 69% and 81% of patients respectively were unaware that chemotherapy was highly unlikely to cure their cancer, again suggesting that clinicians are not comfortable with end of life care discussions (11). A survey of over 4000 US physicians found that one third would not discuss prognosis with a cancer patient who was asymptomatic but had only 4-6 months to live, preferring instead to wait until symptoms developed or there were no more treatments to offer (12).

Our previous study was of a prevalent rather than incident cohort which may have over-represented patients who had longer hospital stays. Likelihood of death was two times higher in medical patients than surgical patients, possibly reflecting the elective nature of most surgical admissions. We did not examine the influence of diagnosis, particularly a cancer diagnosis, as a predictor of death nor did we evaluate the relation between previous hospital admissions and mortality or the mortality risk of hospital inpatients compared to the wider population from which our patients were derived (1,2). The aims of the current study, therefore, were to examine

For peer review only - http://bmjopen.bmj.com/site/about/guidelines.xhtml

an incident rather than a prevalent cohort; to focus on patients admitted as emergencies to medicine; to determine the impact on survival of a cancer diagnosis made within the previous 5 years; to assess whether previous admissions to hospital influenced mortality; and to compare mortality with that of that of an age and sex standardised Scottish general population.

Methods

We included only patients admitted to hospitals in Scotland where the most acute clinical activity occurs: large general hospitals (n=15) and teaching hospitals (n=7). On this occasion we limited our analyses to inpatients admitted as an emergency to medicine between March 18th and March 31st 2015. We defined an inpatient (rather than a day case) as a person who had a Scottish Morbidity Record (SMR01) episode with a discharge date either the day following the admission date, or later. Preliminary checks of numbers of patients and deaths in relation to demographic variables indicated that a 14 day census period resulted in selecting a sufficiently large numbers of admissions and deaths for robust statistical analysis.

In the event of an emergency readmission within the two week census period (n=216), we only counted the patient once. Thus our analysis is based on their first (or only) admission during this period. We refer to this as the census admission and the date of this admission as the census date. We classified patients as having a cancer diagnosis (any malignant neoplasm ICD-10 code C00-C97, excluding non-melanoma skin cancer (NMSC, C44)) (13) if they had an SMR01 record with a cancer diagnosis or a record in the Scottish Cancer Registry database (14) dated five years or less prior to their census date. We also included any cancer diagnoses made in the census stay.

The source of all data related to hospital stays was the Scottish Morbidity Record 01 (SMR01) (15). The measure of deprivation used was the Scottish Index of Multiple Deprivation (SIMD) (16). This is an area based deprivation score, which ranks areas according to a relative measure of deprivation where SIMD 1 represents the 20% most deprived areas in Scotland and SIMD 5 represents the 20% least deprived areas. Linkage to National Records of Scotland (NRS) death records (17) allowed us to follow patients for a year and match time of death to the admission record (18). We limited the analysis to Scottish residents (n=42 persons with invalid or non-Scottish postcodes excluded) and excluded records where record linkage was not possible due to omissions or errors (n=46). We calculated mortality rates for the general Scottish population in 2015 from NRS death records and the NRS mid-year population estimates for 2015.

For peer review only - http://bmjopen.bmj.com/site/about/guidelines.xhtml

Statistical Analysis

We provided statistical summaries in relation to potential risk factors for numbers of admissions and total deaths in the follow-up year, including mortality at seven days, thirty days, three months, six months, nine months and twelve months from the census date. Risk factors were patient demographics (age, sex and deprivation) having a cancer diagnosis (see Methods for definition) and whether admitted as an emergency in the year prior to the census admission. We classified primary diagnoses at census admission and primary causes of deaths using the NRS classification for causes of death in Scotland (19) and documented whether death occurred in a hospital, a care home or other institution, or at a private address.

We produced Kaplan-Meier plots for age, sex, cancer diagnosis, and deprivation to examine differences in survival between groups of patients. Age was grouped into age bands (under 60s and five year age bands above this to 85+) for ease of comparison between younger and older persons and detection of non-linear changes with age. Age groups were the same as the previous study for comparability. We modelled survival in days using multivariate Cox proportional hazards models using R 3.3.2. Follow-up was 366 days as 2016 was a leap year. We censored patients surviving beyond 366 days from the date of their census emergency admission. We conducted univariate analysis to examine the hazard ratio associated with the individual variables. Sex, age, deprivation, cancer diagnosis and admission during the previous year were all included in the multivariate Cox regression to determine whether these factors were independent predictors of survival.

There was some evidence of non-proportionality in the Schoenfield residuals plot for cancer diagnosis, (although the hazard ratio was always greater than one and quantitative test for a linear trend was non-significant). To test the robustness of our model to this slight non-proportionality and to further investigate differences between cancer and non-cancer patients, we repeated the multivariate Cox regression analysis for cancer and non-cancer patients separately. The hazard ratio was non proportional for emergency admissions, in non-cancer patients and all patients combined. The trend fitted a linear function of log time so it was possible to fit an interaction term to account for this. Confidence intervals for the interaction term were computed using the delta method.

Patient and Public Involvement

For peer review only - http://bmjopen.bmj.com/site/about/guidelines.xhtml

Patients and public were not involved in the design or preparation of this study

Results

We identified 10,477 patients with emergency admissions to medicine during the two week period 18th to 31st March 2015 (after exclusions noted in methods). There were more females (52.1%) than males (47.9%). Most patients were 60 years or older (62.6%), and 14.0% were 85 or older. A greater proportion of admissions came from the most deprived areas (SIMD 1, 29.5%) compared to the least deprived areas (SIMD 5, 13.3%). A total of 1,565 (14.9%) patients had been given a cancer diagnosis in the previous five years. Just under half (5067 patients, 48.7%) had required one or more emergency admissions in the year before the census admission.(Table 1)

For peer review only

Table 1: Characteristics of patient cohort and mortality rates

**Age group**	**No. of Admissions**	**%**	**Deaths within 7 days**	**%**	**Deaths within 30 days**	**%**	**Deaths within 3 months**	**%**	**Deaths within 6 months**	**%**	**Deaths within 9 months**	**%**	**Deaths within 1 year**	**%**
0-59	3,915	37.4%	27	0.7%	80	2.0%	179	4.6%	227	5.8%	277	7.1%	309	7.9%
60-64	710	6.8%	12	1.7%	35	4.9%	79	11.1%	104	14.6%	125	17.6%	132	18.6%
65-69	926	8.8%	27	2.9%	64	6.9%	133	14.4%	172	18.6%	207	22.4%	234	25.3%
70-74	1,066	10.2%	39	3.7%	98	9.2%	192	18.0%	229	21.5%	270	25.3%	297	27.9%
75-79	1,175	11.2%	27	2.3%	89	7.6%	203	17.3%	263	22.4%	315	26.8%	351	29.9%
80-84	1,213	11.6%	53	4.4%	125	10.3%	243	20.0%	320	26.4%	376	31.0%	406	33.5%
85+	1,472	14.0%	88	6.0%	202	13.7%	369	25.1%	465	31.6%	560	38.0%	617	41.9%
**Sex**														
Female	5,463	52.1%	145	2.7%	332	6.1%	666	12.3%	857	15.7%	1,031	18.9%	1,138	20.8%
Male	5,014	47.9%	128	2.6%	361	7.2%	732	14.6%	923	18.4%	1,099	21.9%	1,208	24.1%
**SIMD**														
SIMD1 (most)	3,092	29.5%	78	2.5%	204	6.6%	372	12.0%	473	15.3%	564	18.2%	621	20.1%
SIMD2	2,478	23.7%	66	2.7%	155	6.3%	342	13.8%	436	17.6%	519	20.9%	574	23.2%
SIMD3	1,844	17.6%	47	2.5%	119	6.5%	255	13.8%	309	16.8%	374	20.3%	411	22.3%
SIMD4	1,667	15.9%	43	2.6%	121	7.3%	236	14.2%	305	18.3%	370	22.2%	397	23.8%
SIMD5 (least)	1,396	13.3%	39	2.8%	94	6.7%	193	13.8%	257	18.4%	303	21.7%	343	24.6%
**Cancer diagnosis**														
No	8,912	85.1%	187	2.1%	414	4.6%	799	9.0%	1,055	11.8%	1,318	14.8%	1,476	16.6%
Yes	1,565	14.9%	86	5.5%	279	17.8%	599	38.3%	725	46.3%	812	51.9%	870	55.6%
**Emergency admission in previous year**														
No	5,410	51.6%	98	1.8%	250	4.6%	507	9.4%	621	11.5%	747	13.8%	819	15.1%
Yes	5,067	48.4%	175	3.5%	443	8.7%	891	17.6%	1159	22.9%	1383	27.3%	1527	30.1%
**Total**	**10,477**	100.0%	**273**	2.6%	**693**	6.6%	**1,398**	13.3%	**1,780**	17.0%	**2,130**	20.3%	**2,346**	**22.4%**

For peer review only - http://bmjopen.bmj.com/site/about/guidelines.xhtml

Deaths following the census admission

There were 2,346 deaths (22.4% mortality) in the year following the census admission. Table 1 shows the number and percentage of deaths that occurred at different time intervals during the year. Six hundred and ten patients died during the census admission (5.8% of all admissions and 26% of all deaths) while 1,736 of the deaths that occurred during the year did so after the patient had been discharged (74% of all deaths). Overall, men were more likely to die than women (24.1% vs. 20.8%) and this higher mortality was demonstrated within each age group. (Table 1 and Figure 1). Mortality rose steeply with age and was five times higher at one year for patients aged 85 years and over compared to those who were under 60 years of age (41.9% vs. 7.9%). A slightly lower proportion of patients from the most deprived areas (SIMD 1) died during follow-up (20.1%) compared to the less deprived quintiles (ranging from 22.3-24.6%). Cancer patients had higher mortality rate than patients without a cancer diagnosis (55.6% v 16.6% mortality at one year) as did patients who had been admitted during the year prior to the census admission (30.1% v 15.1% mortality) (Table 1).

Figure 1 about here

Cause of death and place of death

Three categories of primary cause of death accounted for almost three quarters of all deaths in the cohort. These were malignant neoplasms (33.8%, with the most common subgroup being cancer of trachea, bronchus and lung); circulatory diseases (22.5%, mainly ischaemic heart disease); and respiratory disease (17.9%, mainly chronic lower respiratory tract disease). The most common place of death was an NHS hospital which accounted for 1,594 (67.9%) of the 2,346 deaths. The remainder of the deaths occurred either at home or other private address (17.8%); or in a care home or other institution (14.3%).

Comparison with the general population

We calculated age standardized mortality rates using the 2013 European Standard Population to take account of the differences in the age distribution of our emergency medical admissions and the Scottish population. Age standardised mortality for men was 122.0 per 1,000 (95% CI 113.0 to 131.0) which was 9 times higher than that of the Scottish population (13.4 per 1,000, 95% CI

For peer review only - http://bmjopen.bmj.com/site/about/guidelines.xhtml

13.3 to 13.6) from which they were derived. Age standardised mortality rates for women in our cohort and the general population were 98.9 per 1,000 (90.9 to 106.9) and 10.0 (95% CI 9.8 to 10.1) respectively, indicating a 10 fold increase in risk. Results for both sexes combined were 110.4 (95% CI 104.4 to 116.5) for the cohort and 11.7 (95% CI 11.6 to 11.8) for the Scottish population, a 9 fold increase in risk.

Cox regression for all patients in the cohort

Gender, age, deprivation, cancer diagnosis and one or more previous emergency admissions all predicted mortality in the univariate analysis (Table 2, Figure 2) but in the multivariate analysis only gender, age, cancer and previous admission were independent predictors of death (Table 2). Men were 1.24 (CI 1.14-1.34) times more likely to die than women after adjusting for other risk factors. Older patients had an increased risk of death: the adjusted hazard ratio for those aged over 85 compared to those aged under 60 was 5.74 (CI 4.99-6.59). Cancer diagnosis was an important independent predictor of death, with nearly four times increase in risk (HR 3.56, CI 3.27-3.88). Emergency admission to hospital in the year prior to the census admission increased risk, although this was non-linear: estimated hazard ratio increased from 1.25 (0.93-1.58) on the census day to 1.67 (CI 1.51-1.82) at 30 days and 2.05 (CI 1.77-2.34) at the end of follow-up. By contrast deprivation did not predict outcome once other factors were taken into account.

Figure 2 about here

ew only

For peer review only - http://bmjopen.bmj.com/site/about/guidelines.xhtml

Table 2: Cox regression analysis for mortality in one year of follow-up, for all patients in the cohort

	No. of deaths	No. of patients	Unadjusted Hazard ratio (95% CI)	p-value	Adjusted Hazard ratio (95% CI)	p-value
**Age**						
<60	309	3,915	1	-	1	-
60-64	132	710	2.51 (2.05-3.08)	<0.001	2.15 (1.75-2.63)	<0.001
65-69	234	926	3.53 (2.98-4.18)	<0.001	2.73 (2.30-3.24)	<0.001
70-74	297	1,066	4.01 (3.42-4.7)	<0.001	2.99 (2.54-3.51)	<0.001
75-79	351	1,175	4.28 (3.68-4.99)	<0.001	3.21 (2.75-3.74)	<0.001
80-84	406	1,213	4.99 (4.31-5.79)	<0.001	4.15 (3.57-4.82)	<0.001
85+	617	1,472	6.60 (5.76-7.57)	<0.001	5.74 (4.99-6.59)	<0.001
**Sex**						
Females	1,138	5,463	1	-	1	-
Males	1,208	5,014	1.18 (1.09-1.28)	<0.001	1.24 (1.14-1.34)	<0.001
**Deprivation**						
SIMD 5 = least deprived	343	1,396	1	-	1	-
SIMD 4	397	1,667	0.97 (0.84-1.12)	0.678	1.00 (0.90-1.12)	0.728
SIMD 3	411	1,844	0.90 (0.78-1.04)	0.146	0.96 (0.85-1.09)	0.676
SIMD 2	574	2,478	0.94 (0.82-1.07)	0.342	0.96 (0.84-1.09)	0.295
SIMD 1= most deprived	621	3,092	0.80 (0.7-0.91)	<0.001	0.93 (0.82-1.07)	0.313
**Cancer diagnosis**						
No	1,476	8,912	1	-	1	-
Yes	870	1,565	4.53 (4.16-4.92)	<0.001	3.56 (3.27-3.88)	< 0.001
**Emergency admission in previous year**						
No	819	5,410	1	-	1	-
Yes	1527	5,067	1.60 (1.24-2.08)	<0.001	1.25 (0.97-1.62)	0.089
Time*Emergency†	-	-	1.08 (1.01-1.14)	0.016	1.09 (1.02-1.15)	0.006

†Time function for interaction was log(t+1).

For peer review only - http://bmjopen.bmj.com/site/about/guidelines.xhtml

Cox regression for cancer and non-cancer patients separately

Men were more likely to die than women in both cancer and non-cancer patient groups. The effect size was similar with adjusted hazards ratios for men compared to women of 1.23 (CI 1.07-1.41) and 1.33 (CI 1.20-1.47) in cancer and non-cancer patients respectively. Absolute mortality increased with age in both groups but was always higher in patients with a cancer diagnosis. Compared to those aged under 60 years non-cancer patients over 85 had a higher adjusted hazard ratio of 10.16 (CI 8.50-12.13) than cancer patients of the same age (adjusted hazard ratio 1.56 (CI 1.23-1.98). This was a consequence of lower absolute mortality in younger patients who did not have cancer (47 per 1000 for non-cancer patients under 60 vs. 463 per 1000 for cancer patients under 60). Compared to patients with no emergency admission in the previous year, non-cancer patients with one or more previous admissions had an adjusted hazard ratio of 1.26 (0.86-1.66) on the census day, 1.36 (0.99-1.77) at 1 day, 1.86 (CI 1.64-2.09) at 30 days and 2.48 (CI 2.07-2.48) at 366 days. Barring the hazard on the admission day itself, this was a higher ratio than observed for cancer patients with one or more previous admissions (adjusted hazard ratio 1.31, CI 1.14-1.51, constant over follow-up time). By contrast deprivation did not predict outcome in either cancer or non-cancer patients. A supplementary table showing the Cox regression for cancer and non cancer patients separately is available on request.

For peer review only

For peer review only - http://bmjopen.bmj.com/site/about/guidelines.xhtml

Discussion

We confirm previous findings in an incident rather than prevalent population of emergency medical admissions to Scottish hospitals in 2015. Over 1 in 5 patients died within a year of their census admission with three quarters of the deaths occurring after rather than during that admission. Mortality rose steeply with age and was five times higher at one year for patients aged 85 years and over compared to those who were under 60. Our new findings are that likelihood of death was more closely related to age and to a cancer diagnosis than it was to gender or social deprivation. Over half of all cancer patients died during the 12 months of follow up. Cancer patients were more than three times likely to die than patients without a cancer diagnosis. Mortality was also significantly higher among patients who had required one or more emergency admissions in the year before the census admission. Age-sex standardized mortality for men and women was nine and ten times higher respectively than the general population from which they were derived.

These findings have important implications for health and social care. Around 550,000 people die in the UK each year. This number is expected to rise to 615,000 deaths per year by 2030 (20). These deaths commonly occur in hospital and are frequently preceded by one or more emergency hospital admissions. There were over 6 million emergency admissions to NHS hospitals in England (21) and Scotland (22) in 2015-16. The continuing rise in emergency admissions to hospital (21) likely reflects an increase in life expectancy that is not always healthy life expectancy. The Office for National Statistics has estimated that between 2013 and 2015 UK men at age 65 could expect to live for a further 18.5 years with 10.3 of these years in good health. The corresponding figures for women aged 65 are 20.9 and 11.1 years respectively. Thus men and women aged 65 can expect to live just over half of their remaining years in good health (23). Similar findings have been reported by European (24) and US investigators (25).

The General Medical Council considers that patients are approaching the end of life when they are likely to die within 12 months. This definition of end of life includes patients whose death is imminent, those with advanced progressive incurable conditions and patients with general frailty and coexisting conditions that mean they are expected to die within 12 months (7). The Gold Standards Framework Proactive Identification Guidance and the Supportive and Palliative Care Indicators Tool may be used to identify people in the latter two groups whose health is deteriorating and who may be entering the last year of life. Both suggest asking the Surprise

For peer review only - http://bmjopen.bmj.com/site/about/guidelines.xhtml

Question ("Would you be surprised if the patient were to die in the next year, months, weeks or days?") and looking for specific clinical indicators of decline relating to the three broad trajectories of illness: cancer, organ failure and frailty (5,26).

There is now a continuum of interventions possible within the scope of modern medicine with growing interest in the integration of palliative care alongside curative treatments, rehabilitation and the management of long term conditions. We know that this is often not addressed during emergency medical admission and that doctors do not always feel comfortable making advance or anticipatory care plans with patients and their families despite recommendations that they should do so (5-9). Up to 26% cancer patients, 59% of those with organ failure and 34% with frailty do not have advance care plans in place before death (27). This means that a request to ask 'difficult questions' relating to cardiopulmonary resuscitation and escalation to high dependency or intensive care when a patient is admitted as an emergency to medicine can sometimes cause unintended distress (28).

If a more modern palliative care orientation is taken, such questions would become subsumed under a broader set of issues relating to the broader goals of the patient. These are eloquently captured by Gawande: What is your understanding of the situation and its potential outcomes? What are your fears and what are your hopes? What are the trade-offs you are willing to make and not willing to make? And what is the course of action that best serves this understanding? (29). All clinicians involved in caring for patients at the end of life have a responsibility to communicate effectively with patients, their families and members of the multi-disciplinary team in order to explore treatment goals and make key decisions (30,31). Good advance care planning in hospital (32) could mean that the next time a frail older patient becomes unwell a course of action ensues which does not result in emergency admission to hospital.

We are aware of limitations to our study. First, if patients emigrated and died abroad during the year of follow up then this would underestimate their mortality. We think this is unlikely to be a source of major imprecision. Second, and more importantly, we recognise that our analysis identifies groups of individuals at high risk of death within one year and that it cannot and should not be used to predict an individual's risk of dying.

In conclusion, we believe these data may help identify groups of patients admitted to hospital as medical emergencies who are at greatest risk of dying not only during admission but also in the following 12 months. Emergency admission to hospital therefore provides an important

For peer review only - http://bmjopen.bmj.com/site/about/guidelines.xhtml

opportunity to make advance care plans if appropriate and if such discussions have not already begun. Ultimately we believe it is wrong to deny the need for an approach at the end of life that might provide care that is more humane and perhaps less costly (33, 34).

For peer review only

For peer review only - http://bmjopen.bmj.com/site/about/guidelines.xhtml

Figure legends

Fig 1: Cumulative deaths as % of census admissions in males and females by age.

Fig 2: Kaplan-Meier survival curves of patient groups by a) sex; b) age group; c) cancer diagnosis; d) SIMD quintile; e) whether or not patient had one or more emergency admissions in the year prior to the census admission.

Copyright

The corresponding author has the right to grant on behalf of all authors and does grant on behalf of all authors, a worldwide licence to the Publishers and its licensees in perpetuity, in all forms, formats and media (whether known now or created in the future), to i) publish, reproduce, distribute, display and store the Contribution, ii) translate the Contribution into other languages, create adaptations, reprints, include within collections and create summaries, extracts and/or, abstracts of the Contribution, iii) create any other derivative work(s) based on the Contribution, iv) to exploit all subsidiary rights in the Contribution, v) the inclusion of electronic links from the Contribution to third party material where-ever it may be located; and, vi) licence any third party to do any or all of the above.

Contributorship

Chris Isles, Emily Moore, Lauren Schofield, Rosalia Munoz-Arroyo and David Clark designed the study. Emily Moore was responsible for record linkage and statistical analyses. Chris Isles wrote the first draft and worked with Alice Radley on the second draft. All authors contributed to the final draft.

Funding statement

This research received no specific grant from any funding agency in the public, commercial or not-for-profit sectors. Professor David Clark is supported by a Wellcome Trust Investigator Award, Grant Number103319/Z/13/Z.

Ethical approval

Not required as no patient identifiable data, in keeping with Scottish Health Boards' policies.

Data sharing statement

Additional data can be accessed in the web appendix. Any queries about source data should be directed to Information Services Division Scotland.

For peer review only - http://bmjopen.bmj.com/site/about/guidelines.xhtml

Competing interests

All authors have completed the ICMJE uniform disclosure form at www.icmje.org/coi_disclosure.pdf and declare: no support from any organisation for the submitted work; no financial relationships with any organisations that might have an interest in the submitted work in the three previous years; no other relationships or activities that could appear to have influenced the submitted work

Transparency declaration

The lead author affirms that the manuscript is an honest, accurate, and transparent account of the study being reported; that no important aspects of the study have been omitted; and that any discrepancies from the study as planned (and, if relevant, registered) have been explained.

For peer review only

For peer review only - http://bmjopen.bmj.com/site/about/guidelines.xhtml

References

1. Clark D, Armstrong M, Allan A, Graham F, Carnon A, Isles C.. Imminence of death among hospital inpatients: prevalent cohort study. *Palliative Medicine* 2014; 28: 474-479.

2. Clark D, Schofield L, Graham F, Gott M, Jarlbaek L, Likelihood of death within one year of a national cohort of hospital inpatients in Scotland. *Journal of Pain and Symptom Management* Accessed at https://dx.doi.org/10.1016/j.jpainsymman.2016.05.07 on 10th October 2017

3. Kellett J, Rasool S, McLoughlin B. Prediction of mortality 1 year after hospital admission. Q J Med 2012; 105:847-853

4. Gott M, Broad J, Zhang X, Jarlbaek L, Clark D. Likelihood of death among hospital inpatients in New Zealand: prevalent cohort study. BMJ Open 2017; 7: e016880. Doi:10.1136/bmjopen-2-17-016880

5. The Gold Standards Framework Proactive Identification Guidance, 2016. Accessed at www.goldstandardsframework.org.uk/PIG 10th September 2017

6. NICE Quality Standard 13. End of life care for adults. First published 28th November 2011. Accessed at www.nice.org.uk/guidance/qs13, 10th September 2017

7. General Medical Council. Treatment and care towards the end of life: good practice in decision making. First published 20th May 2010. Accessed at www.gmc-uk.org/guidance/ethical_guidance/end_of_life_care 10th September 2017

8. NHS England. Actions for end of life care: 2014-2016. First published 11th November 2014. Accessed at www.england.nhs.uk/actions-eolc on 10th September 2017

9. Health Improvement Scotland. Palliative and end of life care. First published March 2013. Accessed at www.healthcareimprovementscotland.org on 10th September 2017

10. Mort D, Lansdown M, Smith N, Protopapa K, Mason M. For better or worse? A report by the National Confidential Enquiry into Patient Outcome and Death, 2008. Accessed at https://www.ncepod.org.uk/SACT-report on 4th October 2017

11. Weeks JC, Catalans PJ, Cronin A et al. Patients' expectations about effects of chemotherapy for advanced cancer. *N Engl J Med* 2012; 367: 1616-25

For peer review only - http://bmjopen.bmj.com/site/about/guidelines.xhtml

12. Keating NL, Landrum MB, Rogers SO et al. Physician factors associated with discussions about end of life care. *Cancer* 2010; 116: 998-1006

13. World Health Organisation, 2010, International Classification of Diseases, Tenth Revision (ICD-10),

14. ISD Scotland, 2010, Scottish cancer registry, http://www.isdscotland.org/Health-Topics/Cancer/Scottish-Cancer-Registry/

15. ISD Scotland, 2017, General Acute Inpatient and Day Case - Scottish Morbidity Record (SMR01), http://www.ndc.scot.nhs.uk/National-Datasets/data.asp?ID=1&SubID=5

16. APS Group Scotland, 2016. Introducing The Scottish Index of Multiple Deprivation 2016, A National Statistics publication for Scotland. Produced for The Scottish Government by APS Group Scotland. Accessed at http://www.gov.scot/Resource/0050/00504809.pdf.

17. National Records of Scotland, 2017. Vital events: Deaths. Accessed at http://www.nrscotland.gov.uk/statisitics-and-data/statistics/statistics-by-theme/vital-events/deaths

18. National Records of Scotland, 2016a. Statistics by theme. Accessed at http://www.nrscotland.gov.uk/statistics-and-data/statistics/statistics-by-theme.

19. National Records of Scotland, 2016b. Vital Events Reference Tables 2015, Section 6: Deaths – Causes. Accessed at https://www.nrscotland.gov.uk/statistics-and-data/statistics/statistics-by-theme/vital-events/general-publications/vital-events-reference-tables/2015/section-6-deaths-causes

20. Office for National Statistics. Underlying death data from the National Population Projection Accuracy Report, released July 2015. Accessed at https://www.ons.gov.uk/peoplepopulationandcommunity/birthsdeathsandmarriages/deaths/adhocs/004886underlyingdeathsdatafromthenationalpopulationprojectionsaccuracyreportreleasedjuly2015 on 7th October 2017

21. NHS Digital. Hospital admitted patient care activity. England, 2015-16. First published 9th November 2016. Accessed at http://www.content.digital.nhs.uk/catalogue/PUB22378 on 11th September 2017

For peer review only - http://bmjopen.bmj.com/site/about/guidelines.xhtml

22. ISD Scotland. Inpatient and day case activity: Emergency admissions by NHS Board and Health and Social Care partnership, October 2016. Accessed at https://www.isdscotland.org/health-topics/hospital-care/inpatient-and-day-case-activity on 7th October 2017

23. Office for National Statistics. Health state life expectancies UK: 2013-2015. First published 29th November 2016. Accessed at https://www.ons.gov.uk/releases/healthstatelifeexpectanciesuk2013to2015 on 11th September 2017

24. Eurostat Statistics Explained. Healthy Life Years Statistics. Accessed at http://ec.europa.eu/eurostat/statistics-explained/index.php/healthy-life-years-statistics.

25. Centers for Disease Control and Prevention. State specific healthy life expectancy at age 65 years – United States, 2007-2009. MMWR 2013; 62: 561-566

26. NHS Lothian. Supportive and Palliative care Indicators Tool (SPICT), last updated 18th April 2017 Accessed at https://www.spict.org on 10th October 2017

27. Tapsfield J, Hall C, Lunan C, McCutcheon H, McLoughlin P, Rhee J et al. Many people in Scotland now benefit from anticipatory Care before they die: an after death analysis and interviews with general practitioners. *BMJ Supportive and Palliative Care* 2016. Accessed online doi:10.1136/bmjspcare-2015-001014

28. The Queen on the Application of David Tracey v Cambridge University Hospitals NHS Foundation Trust, 2014. Neutral Citation Number: (2014) EWCA Civ 822. Accessed at www.judiciary.gov.uk/2014/06 on 28th October 2017

29. Gawande A. Being Mortal: Medicine and what matters in the end. New York: Metropolitan Books, Henry Holt and Company, 2014

30. Finlay S, Wilson M, Isles C. Assessment of frail older adults and end of life. *GM* 2016; 12: 29-33

31. Fritz Z, Slowther A-M, Perkins GD. Resuscitation policy should focus on the patient not the decision. *BMJ* 2017; 356: j813

32. Radley A, Osborne H, McKenzie T, Lightbody C. Do hospital anticipatory care plans improve patient care? An evaluation of end of life care in a District general hospital. Poster presented at Scottish Partnership for Palliative Care, RCPE 2017 Accessed at

For peer review only - http://bmjopen.bmj.com/site/about/guidelines.xhtml

https://www.palliativecarescotland.org.uk/content/annual-conference-2017/ on 9th October 2017

33. Scitovsky A. The high cost of dying: what do the data show? *Millbank Q* 2005; 83: 825-41

34. Gawande A, 2008. Letting go: what should Medicine do when it can't save your life? Accessed at https://www.newyorker.com/magazine/2010/08/02/letting-go-2 on 8th October 2017

For peer review only

For peer review only - http://bmjopen.bmj.com/site/about/guidelines.xhtml

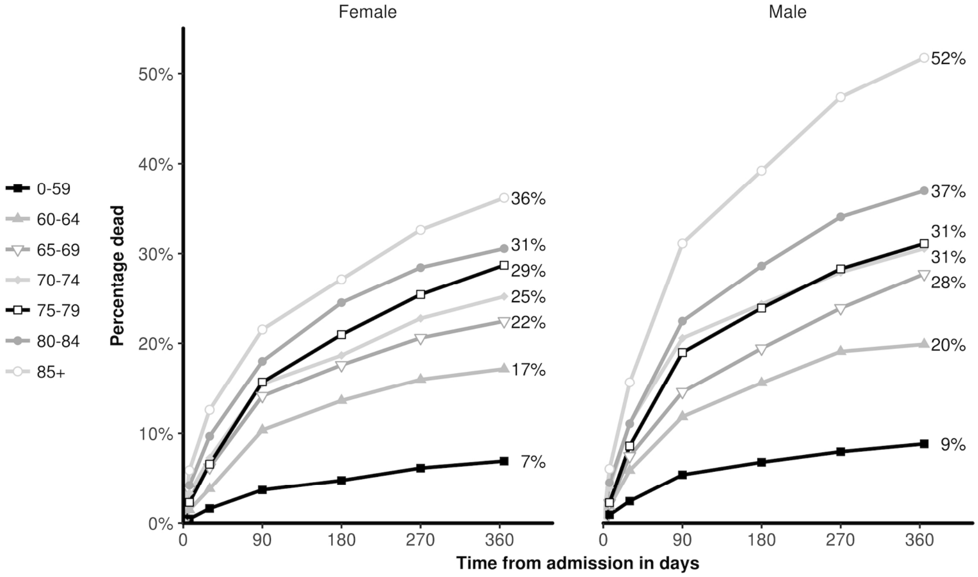

Percentage mortality in females (left panel) and males (right panel) by age and days from admission

199x119mm (300 x 300 DPI)

review only

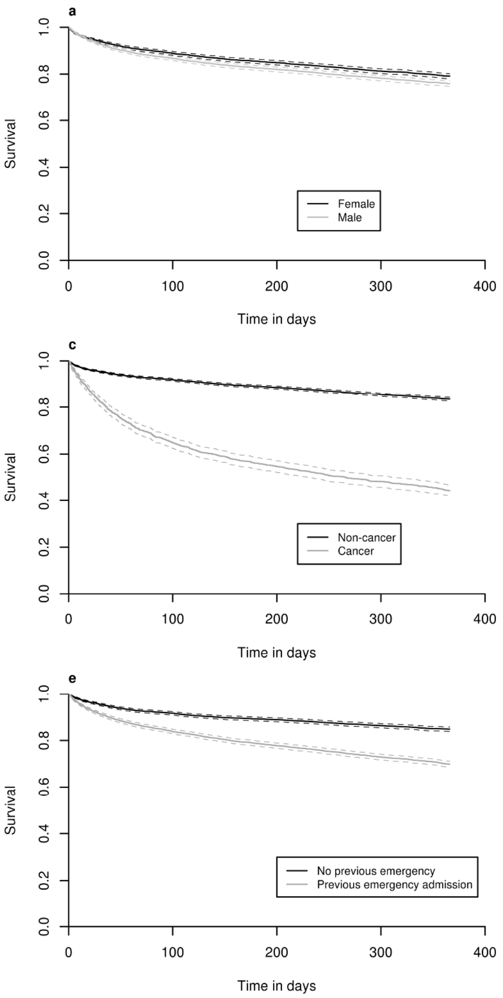

Kaplan-Meier survival curves of patient groups by a) sex; b) age group; c) cancer diagnosis; d) SIMD quintile; e) whether or not patient had one or more emergency admissions in the year prior to the census admission.

133x133mm (600 x 600 DPI)

Supplementary Table: Cox regression analysis for mortality at one year in cancer and non cancer patients

	Cancer patients					Non-cancer patients				
	No. of deaths	No. of patients	Crude mortality rate (per 1,000)	Adjusted hazard ratio (95%CI)	p-value	No. of deaths	No. of patients	Crude mortality rate (per 1,000)	Adjusted hazard ratio (95%CI)	p-value
**Age**										
<60	139	300	463	1	-	170	3,615	47	1	-
60-64	64	118	542	1.27 (0.94-1.71)	0.117	68	592	115	2.52 (1.90-3.34)	<0.001
65-69	124	212	585	1.38 (1.08-1.76)	0.009	110	714	154	3.40 (2.67-4.32)	<0.001
70-74	147	258	570	1.37 (1.09-1.73)	0.008	150	808	186	4.15 (3.33-5.18)	<0.001
75-79	139	264	527	1.20 (0.95-1.52)	0.133	212	911	233	5.23 (4.27-6.40)	<0.001
80-84	125	200	625	1.51 (1.19-1.93)	0.001	281	1,013	277	6.67 (5.51-8.08)	<0.001
85+	132	213	620	1.56 (1.23-1.98)	0.000	485	1,259	385	10.16 (8.50-12.13)	<0.001
**Sex**										
Females	381	742	513	1	-	757	4,721	160	1	-
Males	489	823	594	1.23 (1.07-1.41)	0.003	719	4,191	172	1.33 (1.20-1.47)	<0.001
**Deprivation**										
SIMD 5 = least	140	255	549	1	-	203	1,141	178	1	-
SIMD 4	175	293	597	1.15 (0.92-1.44)	0.208	222	1,374	162	0.99 (0.82-1.20)	0.949
SIMD 3	151	269	561	1.09 (0.86-1.37)	0.486	260	1,575	165	1.00 (0.84-1.21)	0.967
SIMD 2	206	380	542	1.01 (0.82-1.26)	0.911	368	2,098	175	1.11 (0.94-1.32)	0.230
SIMD 1 = most	198	368	538	1.02 (0.82-1.27)	0.856	423	2,724	155	1.16 (0.98-1.38)	0.083
**Emergency admission in previous year**										
No	306	617	496	1	-	513	4,793	107	1	-
Yes	564	948	595	1.31 (1.14-1.51)	<0.001	963	4,119	234	1.26 (0.91-1.73)	0.159
Time*Emergency	-	-	-	-	-	-	-	-	1.12 (1.04-1.21)	0.002

For peer review only

Estimates of hazard ratio for Emergency admission in the previous year for **all patients** at different points in the follow-up

Time (days)	Estimated hazard ratio	LCI	UCI
0	1.25	0.93	1.58
7	1.49	1.27	1.71
30	1.67	1.51	1.83
60	1.77	1.61	1.92
90	1.83	1.66	1.99
120	1.87	1.69	2.05
180	1.93	1.73	2.14
366	2.05	1.77	2.34

Estimates of hazard ratio for Emergency admission in the previous year for **non-cancer patients** at different points in the follow-up

Time (days)	Estimated hazard ratio	LCI	UCI
0	1.26	0.86	1.66
7	1.60	1.30	1.89
30	1.87	1.64	2.09
60	2.02	1.80	2.24
90	2.11	1.88	2.35
120	2.18	1.93	2.44
180	2.29	1.99	2.59
366	2.48	2.07	2.90

For peer review only

STROBE Statement—Checklist of items that should be included in reports of ***cohort studies***

	Item No	Recommendation	Reported on page no
**Title and abstract**	1	(*a*) Indicate the study's design with a commonly used term in the title or the abstract	1
		(*b*) Provide in the abstract an informative and balanced summary of what was done and what was found	2
**Introduction**			
Background/rationale	2	Explain the scientific background and rationale for the investigation being reported	3
Objectives	3	State specific objectives, including any prespecified hypotheses	3
**Methods**			
Study design	4	Present key elements of study design early in the paper	4
Setting	5	Describe the setting, locations, and relevant dates, including periods of recruitment, exposure, follow-up, and data collection	4
Participants	6	(*a*) Give the eligibility criteria, and the sources and methods of selection of participants. Describe methods of follow-up	4
		(*b*) For matched studies, give matching criteria and number of exposed and unexposed	NA
Variables	7	Clearly define all outcomes, exposures, predictors, potential confounders, and effect modifiers. Give diagnostic criteria, if applicable	4,5
Data sources/ measurement	8*	For each variable of interest, give sources of data and details of methods of assessment (measurement). Describe comparability of assessment methods if there is more than one group	4
Bias	9	Describe any efforts to address potential sources of bias	5
Study size	10	Explain how the study size was arrived at	4
Quantitative variables	11	Explain how quantitative variables were handled in the analyses. If applicable, describe which groupings were chosen and why	5
Statistical methods	12	(*a*) Describe all statistical methods, including those used to control for confounding	4,5
		(*b*) Describe any methods used to examine subgroups and interactions	5
		(*c*) Explain how missing data were addressed	3
		(*d*) If applicable, explain how loss to follow-up was addressed	NA
		(*e*) Describe any sensitivity analyses	NA
**Results**			
Participants	13*	(a) Report numbers of individuals at each stage of study—eg numbers potentially eligible, examined for eligibility, confirmed eligible, included in the study, completing follow-up, and analysed	3
		(b) Give reasons for non-participation at each stage	NA
		(c) Consider use of a flow diagram	NA
Descriptive data	14*	(a) Give characteristics of study participants (eg demographic, clinical, social) and information on exposures and potential confounders	5, 6(Table 1), 7, 8
		(b) Indicate number of participants with missing data for each variable of interest	NA
		(c) Summarise follow-up time (eg, average and total amount)	5

For peer review only

For peer review only - http://bmjopen.bmj.com/site/about/guidelines.xhtml

Outcome data	15*	Report numbers of outcome events or summary measures over time	6
Main results	16	(*a*) Give unadjusted estimates and, if applicable, confounder-adjusted estimates and their precision (eg, 95% confidence interval). Make clear which confounders were adjusted for and why they were included	9,10
		(*b*) Report category boundaries when continuous variables were categorized	10, 12
		(*c*) If relevant, consider translating estimates of relative risk into absolute risk for a meaningful time period	NA
Other analyses	17	Report other analyses done—eg analyses of subgroups and interactions, and sensitivity analyses	11,12
**Discussion**			
Key results	18	Summarise key results with reference to study objectives	13
Limitations	19	Discuss limitations of the study, taking into account sources of potential bias or imprecision. Discuss both direction and magnitude of any potential bias	14
Interpretation	20	Give a cautious overall interpretation of results considering objectives, limitations, multiplicity of analyses, results from similar studies, and other relevant evidence	13-14
Generalisability	21	Discuss the generalisability (external validity) of the study results	14
**Other information**			
Funding	22	Give the source of funding and the role of the funders for the present study and, if applicable, for the original study on which the present article is based	16

*Give information separately for exposed and unexposed groups.

Note: An Explanation and Elaboration article discusses each checklist item and gives methodological background and published examples of transparent reporting. The STROBE checklist is best used in conjunction with this article (freely available on the Web sites of PLoS Medicine at http://www.plosmedicine.org/, Annals of Internal Medicine at http://www.annals.org/, and Epidemiology at http://www.epidem.com/). Information on the STROBE Initiative is available at http://www.strobe-statement.org.

For peer review only
